# Effects of minimum unit pricing for alcohol on different income and socioeconomic groups: a modelling study

**DOI:** 10.1016/S0140-6736(13)62417-4

**Published:** 2014-05-10

**Authors:** John Holmes, Yang Meng, Petra S Meier, Alan Brennan, Colin Angus, Alexia Campbell-Burton, Yelan Guo, Daniel Hill-McManus, Robin C Purshouse

**Affiliations:** a School of Health and Related Research, University of Sheffield, Sheffield, UK; b Department of Automatic Control and Systems Engineering, University of Sheffield, Sheffield, UK

## Abstract

**Background:**

Several countries are considering a minimum price policy for alcohol, but concerns exist about the potential effects on drinkers with low incomes. We aimed to assess the effect of a £0·45 minimum unit price (1 unit is 8 g/10 mL ethanol) in England across the income and socioeconomic distributions.

**Methods:**

We used the Sheffield Alcohol Policy Model (SAPM) version 2.6, a causal, deterministic, epidemiological model, to assess effects of a minimum unit price policy. SAPM accounts for alcohol purchasing and consumption preferences for population subgroups including income and socioeconomic groups. Purchasing preferences are regarded as the types and volumes of alcohol beverages, prices paid, and the balance between on-trade (eg, bars) and off-trade (eg, shops). We estimated price elasticities from 9 years of survey data and did sensitivity analyses with alternative elasticities. We assessed effects of the policy on moderate, hazardous, and harmful drinkers, split into three socioeconomic groups (living in routine or manual households, intermediate households, and managerial or professional households). We examined policy effects on alcohol consumption, spending, rates of alcohol-related health harm, and opportunity costs associated with that harm. Rates of harm and costs were estimated for a 10 year period after policy implementation. We adjusted baseline rates of mortality and morbidity to account for differential risk between socioeconomic groups.

**Findings:**

Overall, a minimum unit price of £0·45 led to an immediate reduction in consumption of 1·6% (−11·7 units per drinker per year) in our model. Moderate drinkers were least affected in terms of consumption (−3·8 units per drinker per year for the lowest income quintile *vs* 0·8 units increase for the highest income quintile) and spending (increase in spending of £0·04 *vs* £1·86 per year). The greatest behavioural changes occurred in harmful drinkers (change in consumption of −3·7% or −138·2 units per drinker per year, with a decrease in spending of £4·01), especially in the lowest income quintile (−7·6% or −299·8 units per drinker per year, with a decrease in spending of £34·63) compared with the highest income quintile (−1·0% or −34·3 units, with an increase in spending of £16·35). Estimated health benefits from the policy were also unequally distributed. Individuals in the lowest socioeconomic group (living in routine or manual worker households and comprising 41·7% of the sample population) would accrue 81·8% of reductions in premature deaths and 87·1% of gains in terms of quality-adjusted life-years.

**Interpretation:**

Irrespective of income, moderate drinkers were little affected by a minimum unit price of £0·45 in our model, with the greatest effects noted for harmful drinkers. Because harmful drinkers on low incomes purchase more alcohol at less than the minimum unit price threshold compared with other groups, they would be affected most by this policy. Large reductions in consumption in this group would however coincide with substantial health gains in terms of morbidity and mortality related to reduced alcohol consumption.

**Funding:**

UK Medical Research Council and Economic and Social Research Council (grant G1000043).

## Introduction

Minimum pricing policies for alcohol are under consideration in several countries.^1^, ^2^, ^3^ Most notably, the Scottish Government has passed, but not yet implemented, legislation to introduce a minimum price below which a unit (8 g/10 mL) of pure alcohol cannot be sold to consumers. The UK Government recently withdrew its commitment to introduce the same policy in England and Wales, citing concerns about a lack of evidence of effectiveness and the potential effect on responsible drinkers.^4^ Concerns around the possibility of large effects on individuals with low incomes have also been a prominent feature of the policy debate in both countries.^5^, ^6^ In this report, we aimed to investigate these concerns via a new appraisal of the potential effect of minimum unit pricing across consumption, income, and socioeconomic distributions.

Assessments of implemented minimum price changes for alcohol in Canada have suggested that a minimum unit price would reduce prevalence of deaths and hospital admissions related to alcohol.^7^, ^8^ These conclusions are supported by meta-analyses that show consistent negative associations between alcohol price changes and rates of alcohol consumption and harms including alcohol-related mortality.^9^, ^10^ Previously published UK policy appraisals based on the Sheffield Alcohol Policy Model (SAPM), also suggest that a minimum unit price would have greater effects on heavy drinkers than on moderate drinkers.^11^

A limitation of this evidence is that it does not address whether a minimum unit price has regressive effects by imposing the greatest burden on the poorest groups. Studies examining this question have concluded that regressive effects would, at worst, be small.^12^, ^13^ However, these equity assessments were unbalanced because they focused only on alcohol consumption and spending changes but not effects on health outcomes. This omission is especially important because the lowest socioeconomic groups have the highest risks of alcohol-related harm,^14^ and might accrue the greatest health benefits from the policy. These beneficial effects should be considered alongside spending and consumption changes in any equity judgment.

## Methods

### Study design and data sources

In the SAPM version 2.6, we present policy appraisals for a £0·45 minimum unit price in England in 2014–15, which was previously proposed as the implementation date of the UK Government's policy. We examine the potential policy effects in two ways: first, by definition of five population subgroups based on household income quintiles and, second, by definition of three socioeconomic subgroups based on occupation type. Both analyses estimate policy effects on alcohol consumption and spending and the second also estimates effects on morbidity and mortality related to alcohol consumption. The appendix and technical report contain full methodological details for SAPM version 2.6.^15^

The 2009 General Lifestyle Survey (GLF) provides data for alcohol consumption in adults older than 16 years in England, including details for the mean weekly number of units consumed and highest daily alcohol intake in the survey week for 10 588 respondents.^16^ Consumption data are split by beverage type (beers, cider, wines, spirits, and ready-to-drink beverages [RTDs]). Sampling weights in the GLF dataset were used to adjust for sampling bias and to scale the data to the English population.^16^ We classify drinkers into three groups: moderate drinkers (≤21 units per week for men and ≤14 units per week for women), hazardous drinkers (>21–50 units per week for men and >14–35 units per week for women), and harmful drinkers (>50 units per week for men and >35 units per week for women). Notably, the GLF contains no information on prices paid or purchase location.

The annual UK Living Costs and Food Survey (LCF) uses 14 day diaries to record purchases of products by household members older than 16 years.^17^ Transaction-level data for 2001–02 to 2009 cover 227 933 alcohol transactions by 46 185 individuals. Data for each alcohol purchase includes beverage type, price paid, volume bought, and purchase location (on-trade [eg, in bars or restaurants] *vs* off-trade [eg, in shops]). We adjusted the LCF data to 2011 prices by use of retail price indices specific to alcohol that are available from the Office for National Statistics (ONS).^15^ Sampling weights provided within the LCF dataset were used to correct for sampling bias.^17^ We adjusted sales volumes at different price points for ten beverage types (on-trade and off-trade beer, cider, wine, spirits, and RTDs) to match aggregated England and Wales 2011 sales data purchased from AC Nielsen for off-trade sales and CGA Strategy for on-trade sales. For all policy appraisals, we accounted for inflation by adjusting the £0·45 minimum unit price threshold to 2011 prices separately for each beverage.^15^

We define 54 population subgroups in each dataset on the basis of age, sex, and alcohol consumption level. For our analyses, subgroups are further split by income quintile or nine socioeconomic groups. We linked the GLF and LCF data at subgroup level by assuming that patterns of on-trade and off-trade purchasing in the LCF can be used to apportion individuals' mean weekly consumption in the GLF into the ten beverage types. Because individuals could be purchasing alcohol for other people, sensitivity analyses around this linkage are provided in the appendix.

### Modelling

We used price elasticities for alcohol demand as key inputs to SAPM to model how consumption of a product changes after a price change. For example, a price elasticity of −0·5 suggests that a 1% price increase is associated with a 0·5% decline in consumption. Previous SAPM appraisals used elasticities from cross-sectional analyses of LCF data or from other sources;^11^ however, in SAPM version 2.6, they were derived from a longitudinal analysis.^18^ Ideal individual-level longitudinal panel data for changes in purchasing and prices paid do not exist. However, we developed a pseudo-panel approach by use of repeat cross-sectional LCF 2001–02 to 2009 data. This method defines the same 72 subgroups in each survey year by use of characteristics that are largely stable over time (ie, birth year, sex, and socioeconomic status) and then explicitly analyses the longitudinal time trends in each subgroup for mean weekly purchasing levels and prices paid with a fixed-effects model.^18^ This approach has been applied previously to estimate demand elasticities for various goods,^19^ but not for alcohol until now.

We did separate analyses for the ten beverage types to derive a single population-level 10×10 matrix containing both significant and non-significant own-price and cross-price elasticities. In SAPM, own-price elasticities describe the association between price and consumption for one beverage type whereas cross-price elasticities describe the association between price of one beverage type and consumption for another (table 1). Overall, the new pseudo-panel analysis results in a significant overall elasticity estimate that is similar to that derived from SAPM's previous elasticity approach and from meta-analyses.^10^, ^15^, ^18^ Cross-price elasticities in our study were larger than were those reported from cross-sectional analysis of the LCF, although smaller than those reported in UK time series analyses.^20^ We also report sensitivity analyses that used alternative elasticities, including only own-price elasticities and only significant own-price or cross-price elasticities. Additional sensitivity analyses with subgroup-specific elasticities are presented alongside all elasticity matrices in the appendix.

**Table 1:** Own and cross-price elasticities of demand for ten beverage categories

	**Off-trade beer purchase**	**Off-trade cider purchase**	**Off-trade wine purchase**	**Off-trade spirits purchase**	**Off-trade RTDs purchase**	**On-trade beer purchase**	**On-trade cider purchase**	**On-trade wine purchase**	**On-trade spirits purchase**	**On-trade RTDs purchase**
Off-trade beer price	−0·980%*	−0·189%	0·096%	−0·368%	−1·092%	−0·016%	−0·050%	0·253%	0·030%	0·503%
Off-trade cider price	0·065%	−1·268%*	0·118%	−0·122%	−0·239%	−0·053%	0·093%	0·067%	−0·108%	−0·194%
Off-trade wine price	−0·040%	0·736%*	−0·384%*	0·363%	0·039%	−0·245%	−0·155%	0·043%	−0·186%	0·110%
Off-trade spirits price	0·113%	−0·024%	0·163%	**−0·082%**	−0·042%	0·167%	0·406%	0·005%	0·084%	0·233%
Off-trade RTDs price	−0·047%	−0·159%	−0·006%	0·079%	−0·585%*	−0·061%	0·067%	0·068%	−0·179%*	0·093%
On-trade beer price	0·148%	−0·285%	0·115%	−0·028%	0·803%	−0·786%*	0·867%	1·042%*	1·169%*	−0·117%
On-trade cider price	−0·100%	0·071%	0·043%	0·021%	0·365%	0·035%	−0·591%*	0·072%	0·237%*	0·241%
On-trade wine price	−0·197%	0·094%	−0·154%	−0·031%	−0·093%	−0·276%	−0·031%	−0·871%*	−0·021%	−0·363%
On-trade spirits price	0·019%	−0·117%	−0·027%	−0·280%	−0·145%	−0·002%	−0·284%	0·109%	−0·890%*	0·809%*
On-trade RTDs price	0·079%	0·005%	−0·085%	−0·047%	0·369%	0·121%	−0·394%	−0·027%	−0·071%	**−0·187%**

Cells shows the estimated percentage reduction in quantity purchased that would occur for the column beverage after a 1% increase in price for the row beverage (eg, a 1% increase in off-trade beer prices would lead to a 0·980% decline in off-trade beer purchasing, a 0·19% decline in off-trade cider purchasing and so on). Own price elasticities are shown in bold. RTD=ready-to-drink beverages or alcopops.

* p≤0·05 (exact p values are shown in the appendix). Off-trade beverages are beverages purchased in off-trade outlets (eg, shops) and on-trade beverages are beverages purchased in on-trade outlets (eg, bars and restaurants).

To model the effect of a minimum unit price, we made a conservative assumption that retailers would increase prices only to the minimum threshold and prices above the threshold would be unaffected. We then estimated effects on mean weekly alcohol consumption by combining these implied price changes with the elasticity matrices. This process takes the 2009 GLF population as its baseline and simulates a future GLF sample, in particular estimating a revised average and highest daily consumption for each individual. Because the LCF does not divide spending data into individual drinking occasions, we indirectly modelled effects on highest daily consumption by use of a least-squares linear regression model for each of the three consumption groups, with highest daily consumption estimated as a function of mean consumption, age, and sex.

We modelled the effect of consumption changes on mortality and disease prevalence for 47 chronic and acute conditions attributable wholly or partly to alcohol.^15^ For chronic conditions that are partly attributable to alcohol consumption (eg, alcohol-related cancers), we used functions relating average consumption to health risk as reported elsewhere.^15^ For acute conditions that are partly attributable to alcohol consumption (eg, road traffic accidents), we fitted linear functions relating highest daily consumption to evidence of the alcohol-attributable fraction for the condition.^21^ For chronic conditions (eg, alcoholic liver disease) and acute conditions (eg, alcohol poisoning) that are wholly attributable to alcohol consumption, we fitted functions relating either average or maximum daily consumption to the absolute number of cases reported.^21^ We obtained absolute numbers for baseline condition-specific mortality and morbidity by age and sex subgroups from 2005–06 ONS and Hospital Episodes Statistics data compiled by Jones and colleagues.^22^ We used change in consumption, and thus risk, over time to adjust observed mortality and morbidity rates by applying the potential impact fraction method.^23^ For chronic conditions, a lack of evidence exists regarding the time lag between population-level changes in drinking and associated changes in health harms. We use a linear time lag function of 10 years building up to the full effect, which is consistent with average estimates used elsewhere.^24^

### Model parameters adjustment

This report contains two separate analyses. For the first analysis, we split the population into income quintiles based on respondents' household income, adjusted with the McClement's equivalisation scale to account for differences in disposable income because of household composition.^25^

In the second analysis, we defined subgroups according to the National Statistics Socioeconomic Classification (NS-SEC). Each individual was assigned to one of nine NS-SEC categories or to a missing data category. To estimate baseline mortality risk for each condition by NS-SEC subgroup, we adjusted the population averages from Jones and colleagues^22^ with the latest available ONS data for overall incidence of mortality related to alcohol consumption by socioeconomic group.^26^ Equivalent adjustment data for hospital admissions related to alcohol consumption are not available, so we assumed that the mortality socioeconomic adjustments also apply to morbidity. ONS adjustment data were not available for individuals in “never worked and long-term unemployed” and “missing” subgroups. Therefore, we assigned the baseline mortality risk of NS-SEC Group 7 (the next lowest group) to both subgroups, and report sensitivity analyses around this assumption (appendix). For clarity, when reporting results, we collapse groups into a three-category NS-SEC classification (managerial or professional, intermediate, and routine or manual occupations). Individuals who have never worked, are long-term unemployed, or missing data categories are included in the routine or manual worker group.

We also did sensitivity analyses to examine the effect of application of alternative minimum unit price thresholds and account for under-reporting of alcohol consumption in the GLF survey (appendix).

### Role of the funding source

The sponsor of the study had no role in study design, data collection, data analysis, data interpretation, or writing of the report. The corresponding author had full access to all the data in the study and had final responsibility for the decision to submit for publication.

## Results

Table 2 shows the baseline data and policy appraisal results for the income quintile analysis. Baseline drinking prevalence and average drinking volume increased with increased income. Average weekly consumption in the highest income group was 38% higher than it was in the lowest income group, and 33% of the highest income group drank at hazardous or harmful levels compared with 16% for the lowest (table 2). A similar income gradient was present among moderate drinkers; however, this pattern reversed for harmful drinkers (table 2). Harmful drinkers on a low income consumed more alcohol than did their higher income counterparts.

**Table 2:** Baseline and estimated change in alcohol consumption and spending with a £0·45 minimum unit price, by income and consumption group

		**Overall**	**Quintile 1 (lowest income)**	**Quintile 2**	**Quintile 3**	**Quintile 4**	**Quintile 5 (highest income)**
Sample size		10 588	2130	2335	2121	1980	2022
All drinkers							
	n (%)*	8953 (84%)	1566 (72%)	1858 (78%)	1832 (87%)	1813 (91%)	1884 (93%)
	Baseline consumption (units per week)	14·1	12·7	12·2	13·6	13·9	17·5
	Weekly units purchased at <£0·45	3·3	4·0	3·2	3·2	2·8	2·5
	Percentage of total units purchased at <£0·45	23·2%	31·7%	26·2%	23·2%	19·9%	14·4%
	Baseline spending (£ per year)	£633	£492	£505	£620	£647	£849
	Change in consumption (%)	−1·6%	−4·4%	−2·4%	−1·7%	−0·8%	−0·1%
	Change in consumption (yearly units per drinker)	−11·7	−29·3	−15·1	−12·4	−5·5	−0·8
	Change in spending (£ per drinker per year)	£2·12	−£1·89	£0·11	£1·84	£3·22	£6·10
Moderate drinkers							
	n (%)*	6545 (62%)	1235 (57%)	1432 (60%)	1389 (66%)	1299 (66%)	1190 (60%)
	Baseline consumption (units per week)	5·5	4·6	4·5	5·5	5·8	6·8
	Weekly units purchased at <£0·45	0·7	0·8	0·7	0·6	0·5	0·4
	Percentage of total units purchased at <£0·45	12·5%	18·4%	15·8%	11·6%	8·4%	5·5%
	Baseline spending (£ per year)	£286	£202	£207	£283	£317	£412
	Change in consumption (%)	−0·6%	−1·6%	−1·2%	−0·5%	−0·3%	0·2%
	Change in consumption (yearly units per drinker)	−1·6	−3·8	−2·8	−1·6	−0·8	0·8
	Change in spending (£ per drinker per year)	£0·78	£0·04	£0·34	£0·79	£0·85	£1·86
Hazardous drinkers							
	n (%)*	1851 (18%)	227 (11%)	337 (15%)	346 (16%)	405 (20%)	536 (26%)
	Baseline consumption (units per week)	27·2	26·9	26·7	26·9	27·6	27·5
	Weekly units purchased at <£0·45	5·3	7·9	6·0	5·3	4·0	2·7
	Percentage of total units purchased at <£0·45	19·5%	29·2%	22·6%	19·7%	14·4%	9·8%
	Baseline spending (£ per year)	£1180	£1019	£1076	£1146	£1219	£1298
	Change in consumption (%)	−0·6%	−3·0%	−1·8%	−0·7%	0·1%	0·4%
	Change in consumption (yearly units per drinker)	−9·1	−41·7	−24·8	−9·5	1·7	5·4
	Change in spending (£ per drinker per year)	£8·67	£2·60	£1·56	£6·80	£13·19	£12·89
Harmful drinkers							
	n (%)*	557 (5%)	104 (5%)	89 (4%)	97 (5%)	109 (5%)	158 (8%)
	Baseline consumption (units per week)	71·4	75·9	74·4	76·8	65·5	67·1
	Weekly units purchased at <£0·45	21·8	30·8	25·2	23·8	18·4	13·6
	Percentage of total units purchased at <£0·45	30·5%	40·6%	33·9%	31·0%	28·1%	20·3%
	Baseline spending (£ per year)	£2862	£2685	£2892	£3317	£2709	£2751
	Change in consumption (%)	−3·7%	−7·6%	−4·3%	−4·0%	−2·8%	−1·0%
	Change in consumption (yearly units per drinker)	−138·2	−299·8	−165·0	−160·3	−97·1	−34·3
	Change in spending (£ per drinker per year)	−£4·01	−£34·63	−£8·67	−£0·11	−£6·08	£16·35

* Numbers refer to absolute sample size, percentages refer to the sample size after survey weights have been applied.

**Figure 1:**
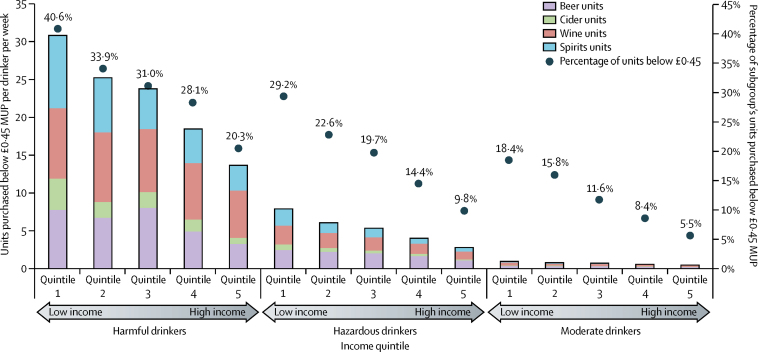
Absolute numbers and proportion of total alcohol units purchased for less than a £0·45 MUP by drinker and income quintile and by beverage type Absolute numbers exclude units of on-trade alcohol (eg, units purchased in bars or restaurants) and off-trade (eg, shop-bought) ready-to-drink beverages because these beverages account for less than 1% of each subgroup's units purchased for less than £0·45. MUP=minimum unit price.

Figure 1 shows the volume of alcohol sold at less than a £0·45 per unit threshold. Moderate drinkers purchased a mean of 0·7 units per week at prices lower than the threshold, with hazardous drinkers purchasing 5·3 such units. By contrast, harmful drinkers purchased on average more than 30 times more units for less than £0·45 than did moderate drinkers. Furthermore, the amounts varied substantially by income, with harmful drinkers on the lowest incomes purchasing 30·8 units per week at less than the minimum unit price (40·6% of their alcohol) whereas harmful drinkers in the highest income group purchased 13·6 units at less than this price (20·3% of their purchases). Almost all (>99%) units bought for less than the minimum unit price were purchased in the off-trade (ie, supermarkets or shops) in each subgroup.

**Figure 2:**
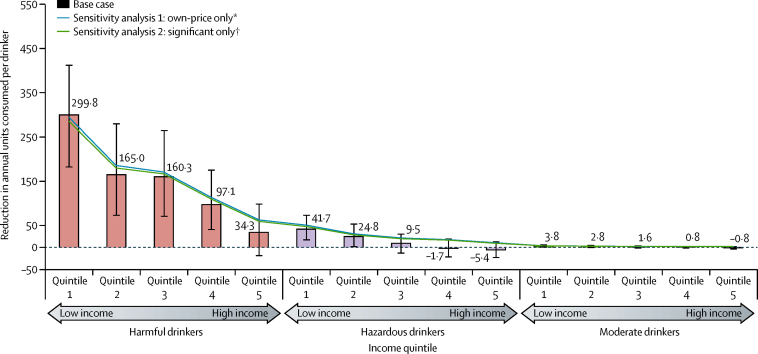
Estimated reductions in annual units consumed per drinker by income quintile and consumption group for a £0·45 minimum unit price, with sensitivity analyses Error bars show 95% CIs calculated with a probabilistic sensitivity analysis. Details of the sensitivity analyses are shown in the appendix. *Cross-price elasticities assumed to be zero. †Non-significant own-price or cross-price elasticities assumed to be zero.

In our analysis, a £0·45 minimum unit price led to an overall estimated reduction in population alcohol consumption of −1·6% (−12 units per drinker per year; table 2, figure 2). Substantial heterogeneity existed in the results between groups, with much larger reductions in harmful drinkers than in moderate or hazardous drinkers (table 2). Consumption in the lowest income group was estimated to decrease by more than 4% and smaller reductions were seen as incomes increased; especially in the upper two quintiles in which the decrease was estimated to be less than −1%. Consumption in harmful drinkers was estimated to decrease in all income groups with by far the largest decrease in individuals with the lowest incomes, although smaller decreases were also estimated for the highest income group (table 2). The estimated effect on moderate drinkers was much smaller than it was for heavier drinkers with less variation across the income distribution (table 2). We noted much the same results in sensitivity analyses (figure 2, appendix).

Most of the total reduction in consumption occurred within the 5·3% of the population who were harmful drinkers (74·0% of the total reduction), with the two lowest income quintiles contributing 66·7% of the overall total. Estimated policy effects for consumer spending on alcohol were related to the estimated change in consumption, and also depend on the beverage preferences of each population subgroup and the own-price and cross-price elasticities associated with those beverages. Most changes in spending were modest in absolute terms and range from a reduction of almost £35 per year for harmful drinkers in the lowest income group (from a baseline of £2685) to an increase of £16·35 for harmful drinkers in the highest income group (from a baseline of £2751). Effects on moderate drinkers were much smaller, with an annual increase in spending of £0·04 in the lowest income group compared with an increase of £1·86 in the highest income group.

In our analysis split by socioeconomic groups, we noted more substantial reductions in consumption in routine or manual groups than in intermediate groups, which in turn had greater reductions than in managerial or professional groups (table 3). 10 years after implementation, a £0·45 minimum unit price was estimated to lead to substantive annual reductions in mortality and illness related to alcohol consumption (table 4). For each outcome measure, the relative reductions were at least twice as large for harms occurring in routine or manual worker households compared with intermediate, managerial, or professional households. Routine or manual worker households made up 41·7% of the population but accrued proportionately more of the harm reductions (>80% of reductions in deaths, hospital admissions, and quality-adjusted life years lost; table 4). The overall 10-year reduction from a £0·45 minimum unit price in health-care costs related to alcohol consumption and health-related quality of life was estimated to be £2·6 billion.

**Table 3:** Baseline and estimated change in alcohol consumption and spending with a £0·45 minimum unit price, by socioeconomic and consumption group

	**Routine or manual**	**Intermediate**	**Managerial or professional**
**Drinkers**			
n (%)*	3505 (79%)	1491 (83%)	3957 (90%)
Baseline consumption (units per week)	13·3	13·5	15·1
Change in consumption (%)	−3·2%	−1·2%	−0·5%
**Moderate drinkers**			
n (%)*	2678 (60%)	1093 (61%)	2774 (64%)
Baseline consumption (units per week)	4·9	5·1	6·2
Change in consumption (%)	−1·2%	−0·6%	0·0%
**Hazardous drinkers**			
n (%)*	622 (14%)	314 (17%)	915 (21%)
Baseline consumption (units per week)	27·1	27·0	27·3
Change in consumption (%)	−1·8%	−0·8%	0·2%
**Harmful drinkers**			
n (%)*	205 (5%)	84 (5%)	268 (6%)
Baseline consumption (units per week)	75·7	71·3	67·8
Change in consumption (%)	−6·3%	−2·5%	−1·7%

Data for 10588 survey respondents.

* Numbers refer to absolute sample size, percentages refer to the sample size after survey weights have been applied.

**Table 4:** Health harm reductions in year 10 with a £0·45 minimum unit price, by socioeconomic group

		**Population**	**Routine or manual**	**Intermediate**	**Managerial or professional**
Percentage of overall population*		10 588 (100%)	4407 (42%)	1791 (17%)	4390 (42%)
Baseline health harms in year 10					
	Deaths	9730	5550	1560	2620
	Chronic illnesses (thousands)	309	162·5	54·8	92·0
	Acute illnesses (thousands)	156	92·0	27·3	36·7
	Hospital admissions (thousands)	763	413·3	133·9	215·7
	QALYs (thousands)	925	552·7	159·7	213·3
Baseline cumulative discounted costs: years 1–10					
	Health-care costs (millions)	£18 054	£10 436	£3182	£4436
	QALY value (millions)	£55 490	£33 164	£9585	£12 741
	Total costs (millions)	£73 544	£43 601	£12 766	£17 177
Annual changes in year 10					
	Deaths	−860	−710	−70	−90
	Reduction in deaths (%)	−8·9%	−12·7%	−4·6%	−3·3%
	Chronic illnesses (thousands)	−12·4	−10·5	−0·9	−1·0
	Reduction in chronic illnesses (%)	−4·0%	−6·5%	−1·6%	−1·1%
	Acute illnesses (thousands)	−3·3	−3·2	−0·2	0·1
	Reduction in acute illnesses (%)	−2·1%	−3·5%	−0·7%	0·3%
	Hospital admissions (thousands)	−29·9	−25·7	−2·1	−2·1
	Reduction in hospital admissions (%)	−3·9%	−6·2%	−1·6%	−1·0%
	QALYs (thousands)	−33·3	−29·0	−2·5	−1·8
	Reduction in QALYs (%)	−3·6%	−5·2%	−1·6%	−0·8%
	Share of reduction in deaths (%)	100·0%	81·8%	8·3%	9·9%
	Share of reduction in chronic illnesses (%)	100·0%	84·8%	7·2%	8·0%
	Share of reduction in acute illnesses (%)	100·0%	96·9%	6·1%	−3·0%
	Share of reduction in hospital admissions (%)	100·0%	86·0%	7·0%	7·0%
	Share of reduction in total QALYs saved (%)	100·0%	87·1%	7·5%	5·4%
Cumulative discounted cost changes: years 1–10					
	Health-care costs (millions)	−£561	−£494	−£40	−£27
	Reduction in health-care costs (%)	−3·1%	−4·7%	−1·3%	−0·6%
	QALY value (millions)	−£1996	−£1737	−£150	−£108
	QALY value (%)	−3·6%	−5·2%	−1·6%	−0·8%
	Total cost savings (millions)	−£2557	−£2232	−£190	−£135
	Total cost savings (%)	−3·5%	−5·1%	−1·5%	−0·8%
	Share of total costs saved (%)	100·0%	87·3%	7·4%	5·3%

QALY=quality-adjusted life-year.

* Numbers refer to absolute sample size, percentages refer to the sample size after survey weights have been applied.

**Figure 3:**
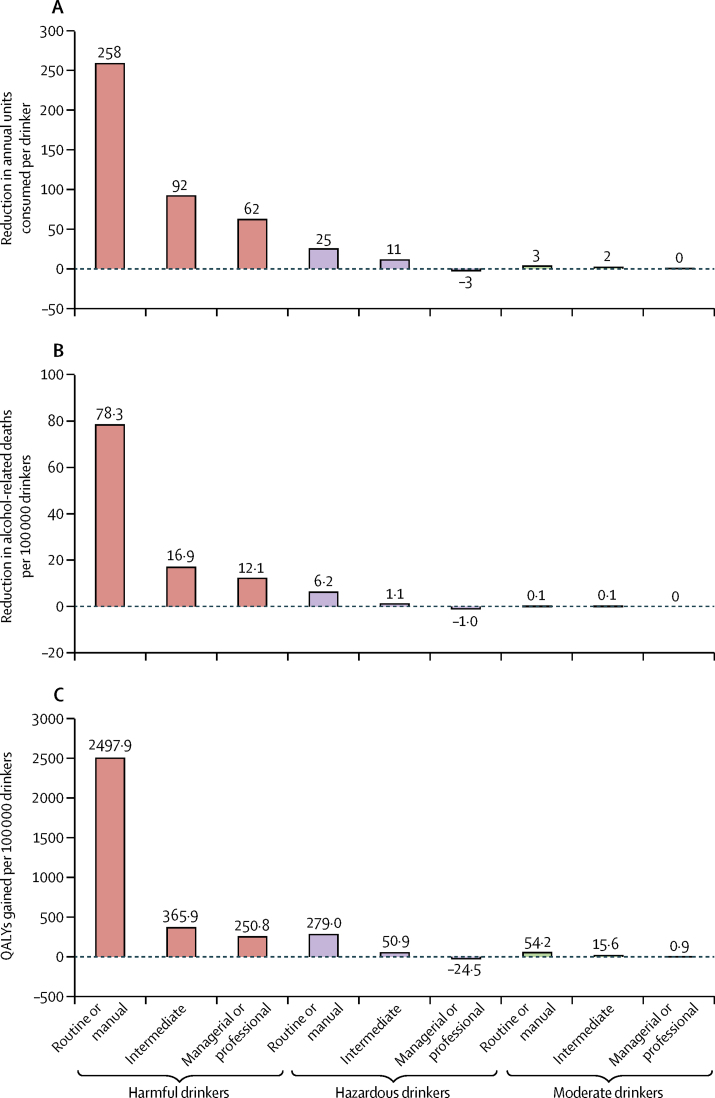
Estimated consumption and harm changes for a £0·45 minimum unit price, by socioeconomic and consumption group (A) Change in annual number of units consumed per drinker. (B) Change in number of deaths related to alcohol per 100 000 drinkers in year 10. (C) Cumulative QALYs gained per 100 000 drinkers in years 1–10 (discounted). QALY=quality-adjusted life-year.

The minimum unit price policy would primarily reduce alcohol consumption and reduce harm (in terms of mortality and lower quality of life) in harmful drinkers and individuals of lower socioeconomic status (figure 3). The estimated reduction in mortality related to alcohol consumption in harmful drinkers was six-times greater for individuals in routine or manual households than it was in individuals in managerial or professional households and the gain in quality-adjusted life-years was ten-times greater (figure 3). Because the reduction in consumption was only four-times greater between these groups, this difference suggests that reductions in consumption in harmful drinkers in the lowest socioeconomic groups result in greater harm reductions than in higher socioeconomic groups, especially for morbidity related to alcohol consumption.

Results of the sensitivity analyses are shown in the appendix. The pattern of results across population subgroups was consistent with our base-case analysis, despite variations in the size of effects when different parameters or assumptions were applied.

## Discussion

Our policy appraisals suggest that introduction of a £0·45 minimum unit price would have substantially different effects across consumption, income, and socioeconomic groups. These differences are driven by the alcohol consumption and purchasing patterns of population subgroups, the price elasticities of the different alcoholic beverages they purchase, and each group's risk of harm related to alcohol consumption. Moderate drinkers, including those with low incomes, would be little affected by the policy because they purchase only small quantities of alcohol at less than the proposed £0·45 minimum unit price threshold. Both non-drinking and moderate consumption are more prevalent in low income groups, meaning large proportions of these groups would not be substantially affected by the policy. Effects would be much more striking for harmful drinkers. The model estimates that harmful drinkers with the lowest incomes would reduce their consumption the most, while consumption in harmful drinkers with high incomes would also reduce. Notably, the estimated health benefits from the policy are also unequally distributed among socioeconomic groups. Most health gains occur in harmful drinkers in the poorest routine or manual worker groups, suggesting that the policy could contribute substantially to the reduction of health inequalities.

Absolute spending changes in all subgroups were small, with most groups showing slight increases; however, small spending reductions were noted for some low-income groups. Spending reductions occur when drinkers purchase beverages with high own-price elasticities and predominantly negative cross-price elasticities, as is the case for low income drinkers (table 1, figure 1). This finding suggests that drinkers on a low income are less able to absorb price increases and their resultant behavioural changes (eg, switching between beverage types) lead to small overall reductions in spending across all products.

Our results provide the most comprehensive analysis to date of the equity implications of a minimum unit price and, in particular, provide to our knowledge the first estimates of how effects on alcohol-related mortality and morbidity can vary by socioeconomic status (panel). Limitations include the indirect modelling of changes in highest daily consumption, which is important because occasions of heavy drinking might be less responsive to price changes,^29^ and not accounting for potential secondary policy effects (eg, increased illicit alcohol trading or market restructuring). Data used for adjustment of baseline health risks by socioeconomic status were only available for overall mortality related to alcohol consumption rather than for individual conditions or for morbidity; thus, harm reductions might have been incorrectly apportioned across socioeconomic groups. Recent meta-analyses have noted different risk curves for mortality compared with morbidity; for example, in liver cirrhosis.^30^ Equivalent meta-analyses are not available for most of the 47 conditions modelled by SAPM, and for consistency we do not use separate mortality and morbidity risk functions for chronic conditions that are partly attributable to alcohol consumption. If new evidence shows equivalent patterns for other conditions, the model structure can incorporate these changes. Further research providing evidence specific to each condition by socioeconomic group and separating mortality from morbidity would be beneficial. In general, SAPM's base case uses conservative assumptions when faced with data limitations or uncertainty, and comparison of results for the Canadian adaptation of SAPM with assessments of minimum price changes in Canada suggests that the model might underestimate the effects of minimum unit price.^7^, ^8^, ^31^ Critiques of SAPM have been produced by consultants commissioned by the alcohol industry^32^, ^33^ and a free-market-oriented think tank.^6^ Their main arguments have been addressed in a detailed rebuttal.^34^

Panel **Research in context**

**Systematic review**

We searched PubMed and Google Scholar up to July, 2013, for reports published in English and contacted experts to identify reports detailing effects related to income groups in alcohol pricing policy. Search terms included income, socioeconomic, poor, poverty, or disadvantage and alcohol, pricing, or policy. We prioritised policy appraisal and evaluation studies. Analyses of alcohol-demand elasticities generally show greater price elasticities for lower income groups than for high-income ones.^27^ More detailed analyses of the equity implications of interventions for alcohol pricing were provided in three studies, but these reports were restricted to considerations of alcohol purchasing and consumption as outcomes. An analysis^13^ of purchasing patterns of alcohol with data from 2 week spending diaries in Scotland reported that the predicted proportion of households purchasing alcohol at less than the minimum unit price and the predicted quantity of such purchases were higher for harmful drinkers than they were for moderate drinkers. Moreover, within each consumption group, a modest income gradient was reported with the lowest income groups most likely to purchase alcohol at less than the minimum unit price. A second study^12^ estimated changes in alcohol spending on the basis of a uniform price elasticity of −0·5 for all products (ie, a 1% increase in prices would lead to a 0·5% fall in consumption) and analysis of patterns in alcohol purchasing from British continuous spending diaries (ranging from 84 days to 1 year). The study concluded that a minimum unit price would be mildly regressive because estimated percentage increases in grocery spending were negatively associated with income. Daley and colleagues^28^ estimated increases in alcohol duty paid per head for a hypothetical $0·25-per-drink tax increase with a US consumption survey and a uniform price elasticity of −0·51 for all alcohol products. The group reported that increases in duty paid were associated positively with income for low-risk drinkers but negatively associated with income for high-risk drinkers. No empirical studies were identified that examined differential effects of alcohol price policies on health outcomes by income group.

**Interpretation**

Our findings are not directly comparable with previous studies because our detailed analyses consider drinkers' preferences for different products and purchasing locations and changes to these preferences after price changes. We also examine a wider range of outcome measures (alcohol consumption, spending, and multiple health harm measures) and provide analyses for both income and socioeconomic status. Dependent on the outcome in question, we estimate that a minimum unit price has both progressive (spending and health harms) and regressive (consumption) effects and these need to be balanced in any judgment of the equity implications of the policy. However, our findings are in line with the previous evidence that effects on moderate drinkers are small and the substantive effects of the policy are restricted to individuals drinking at high rates for all income groups.

A key strength of SAPM is its provision of estimated policy effects for several outcomes across several subgroups. Previous analyses of the equity implications of public health interventions related to price, such as alcohol or tobacco taxation, rarely account for changes in public health outcomes and are instead based on analyses of changes in spending or consumption.^28^, ^35^ For example, the Institute for Fiscal Studies has argued that a minimum unit price is mildly regressive on the basis of analyses in which potential spending changes show small negative associations with income. SAPM's more complex elasticity model suggests that a minimum unit price is mildly progressive on this measure; however, it also estimates large harm reductions for low socioeconomic groups, suggesting minimum unit price is a progressive policy in terms of reduction of health inequalities. Furthermore, although average consumption reductions would be greatest among drinkers with the lowest incomes, our findings concur with Ludbrook and colleagues,^13^ who suggest that these differences are only substantive for individuals consuming large quantities of alcohol and note that fewer heavy drinkers are in low-income groups than in high-income groups. If, as argued by some commentators,^35^ reductions in consumption itself induced by policy are negative effects, then our results suggest a minimum unit price has a mixture of regressive (consumption) and progressive (health outcomes) effects. This mixture of effects provides no support for the UK Government's concerns about the effect of a minimum unit price on responsible drinkers and suggests that claims of the policy being regressive need substantial qualification.

Overall, moderate drinkers, irrespective of income, would only be affected marginally by a minimum unit price policy. The policy would mainly affect harmful drinkers. Moreover, because harmful drinkers on low incomes purchase most alcohol at less than the minimum unit price, this group would be more affected than would individuals on higher incomes. Policy makers need to balance large reductions in consumption by harmful drinkers on a low income against the greater health gains that could be made in this group from reduced morbidity and mortality related to alcohol consumption.

**This online publication has been corrected. The corrected version first appeared at thelancet.com on February 13, 2014**
