# Supplementary material for: Effects of minimum unit pricing for alcohol on different income and socioeconomic groups: a modelling study
Source: Lancet. 2014 May 10;383(9929):1655–64. doi: 10.1016/S0140-6736(13)62417-4 (PMC4018486; doi:10.1016/S0140-6736(13)62417-4)
Supplement: Supplementary appendix [file mmc1.pdf]

THE LANCET

Supplementary appendix

This appendix formed part of the original submission and has been peer reviewed. We post it as supplied by the authors.

Supplement to: Holmes J, Meng Y, Meier PS, et al. Effects of minimum unit pricing for alcohol on different income and socioeconomic groups: a modelling study. *Lancet* 2014; published online Feb 10. http://dx.doi.org/10.1016/S0140-6736(13)62417-4.

Online Appendix: Equity implications of minimum unit pricing for alcohol: impacts on alcohol consumption, spending and population health by socio-economic group

This appendix presents (1) additional methodological information, (2) new sensitivity analyses using the current version of the Sheffield Alcohol Policy Model (SAPM v2.6), which is used for the base case analyses reported in the main article and (3) a set of relevant sensitivity analyses that were previously published by the University of Sheffield in technical reports and which are reproduced here for ease of reference. For these analyses, we state the model version used and the source report.

Table of contents

1. Additional methods detail 2
   1.1. Deriving socioeconomic classification-specific mortality and morbidity rates 2
      1.1.1. Step 1: Baseline age, gender and health condition-specific mortality and morbidity are extracted from a previous report for use as the basis for adjustments........ 2
      1.1.2. Step 2: Derivation of age, gender and SEC-specific adjustment factors for alcohol-related mortality rates. 7
      1.1.3. Step 3: Estimation of SEC-specific alcohol-related mortality rates using SAPM. 9
      1.1.4. Step 4: Modifying adjustment factors to account for SEC-related differences in drinking patterns already modelled in SAPM. 10
      1.1.5. Step 5: Applying mortality adjustment factors to morbidity data. 14
2. New sensitivity analyses 16
   2.1. Probabilistic sensitivity analysis 16
   2.2. Alternative price elasticity matrices 16
   2.3. Alternative price thresholds 24
   2.4. Never worked and long-term unemployed - NS-SEC Group 8 25
3. Previous sensitivity analyses 27
   3.1. Effects of accounting for underestimation of alcohol consumption in self-report population surveys (source: Scottish adaptation of SAPM, second update)[7] 27
   3.2. Effects of making alternative assumption regarding who consumes purchased alcohol (source: version 2.0 of English SAPM)[9] 27
4. References 30

1. Additional methods detail

1.1. Deriving socioeconomic classification-specific mortality and morbidity rates

Investigation and adjustment of SAPM's subgroup-specific mortality and morbidity rates to account for variation in risk across socioeconomic (SEC) groups was undertaken in five steps, as detailed below.

1.1.1. Step 1: Baseline age, gender and health condition-specific mortality and morbidity are extracted from a previous report for use as the basis for adjustments

Tables A1-A4 show the age and gender-specific mortality and morbidity rates for the 48 alcohol-related health conditions modelled in SAPM. These values are calculated using absolute England mortality and person-specific hospital admissions figures for 2005 and 2005/6 respectively as reported in Tables 15-16 of Jones et al.[1] These are the most up-to-date age, gender and condition-specific data available. Rates were obtained by dividing the absolute numbers by corresponding population statistics for England in 2005.[2] Health conditions are categorised as either wholly alcohol-attributable (e.g. alcohol poisoning) or partially alcohol-attributable (e.g. ischaemic heart disease). For partially attributable conditions, the rates in Tables A1-A4 are overall rates, not alcohol-attributable rates.

Table A1: Male alcohol-related mortality rates (per 100,000) for England in 2005

Condition	Type	16-17	18-24	25-34	35-44	45-54	55-64	65-74	75+
Alcohol-induced pseudo-Cushing's syndrome	Wholly attributable	0.0	0.0	0.0	0.0	0.0	0.0	0.0	0.0
Mental and behavioural disorders due to use of alcohol	Wholly attributable	0.1	0.1	0.7	2.0	2.7	3.3	2.4	1.4
degeneration	Wholly attributable	0.0	0.0	0.0	0.0	0.1	0.1	0.0	0.2
Alcoholic polyneuropathy	Wholly attributable	0.0	0.0	0.0	0.0	0.0	0.0	0.0	0.0
Alcoholic myopathy	Wholly attributable	0.0	0.0	0.0	0.0	0.1	0.0	0.0	0.0
Alcoholic cardiomyopathy	Wholly attributable	0.0	0.0	0.1	0.3	0.6	0.7	0.8	0.1
Alcoholic gastritis	Wholly attributable	0.0	0.0	0.0	0.0	0.0	0.0	0.0	0.0
Alcoholic liver disease	Wholly attributable	0.0	0.1	2.6	11.8	27.0	28.3	20.4	8.5
Chronic pancreatitis	Wholly attributable	0.0	0.0	0.1	0.1	0.4	0.3	0.1	0.1
Ethanol poisoning	Wholly attributable	0.0	0.0	0.0	0.2	0.1	0.1	0.0	0.0
Methanol poisoning	Wholly attributable	0.0	0.0	0.0	0.0	0.0	0.0	0.0	0.0
Toxic effect of alcohol, unspecified	Wholly attributable	0.0	0.1	0.4	0.5	1.0	0.4	0.4	0.1
Accidental poisoning by exposure to alcohol	Wholly attributable	0.0	0.1	0.3	0.7	1.1	0.5	0.4	0.1
Malignant neoplasm of lip, oral cavity and pharynx	Partially attributable	0.2	0.1	0.4	0.8	4.1	10.0	14.5	17.6
Malignant neoplasm of oesophagus	Partially attributable	0.0	0.0	0.1	1.3	9.0	27.7	59.1	111.0
Malignant neoplasm of colon	Partially attributable	0.1	0.1	0.5	1.3	5.7	19.2	57.5	149.2
Malignant neoplasm of rectum	Partially attributable	0.0	0.0	0.2	0.8	3.6	12.0	30.5	64.0
Malignant neoplasm of liver and intrahepatic bile ducts	Partially attributable	0.1	0.1	0.3	0.7	3.7	8.9	23.1	42.5
Malignant neoplasm of larynx	Partially attributable	0.0	0.0	0.0	0.2	1.2	4.1	6.1	14.1
Malignant neoplasm of breast	Partially attributable	0.0	0.0	0.0	0.0	0.1	0.4	0.7	2.2
Diabetes mellitus (typeII)	Partially attributable	0.0	0.0	0.0	0.1	0.4	1.1	5.9	24.5
Epilepsy and status epilepticus	Partially attributable	1.0	1.2	1.7	3.0	3.0	2.5	2.6	5.6
Hypertensive diseases	Partially attributable	0.0	0.0	0.3	0.9	2.9	6.8	15.5	60.5
Ischaemic heart disease	Partially attributable	0.1	0.3	2.6	17.1	70.5	189.5	491.1	1666.3
Cardiac arrhythmias	Partially attributable	0.1	0.3	0.1	0.4	0.4	0.8	5.4	46.9
haemorrhagic stroke	Partially attributable	0.6	0.5	1.3	3.5	9.6	15.0	30.8	90.9
Ischaemic stroke	Partially attributable	0.1	0.1	0.4	1.0	4.6	17.9	83.5	550.1
Oesophageal varices	Partially attributable	0.0	0.0	0.0	0.0	0.1	0.2	0.3	0.6
Gastro-oesophageal laceration-haemorrhage syndrome	Partially attributable	0.0	0.0	0.0	0.1	0.1	0.2	0.1	0.6
Unspecified liver disease	Partially attributable	0.0	0.0	0.3	1.9	6.5	8.5	10.8	12.9
heart failure	Partially attributable	0.4	0.3	0.8	1.2	2.6	6.1	21.6	197.8
Cholelithiasis	Partially attributable	0.0	0.0	0.0	0.0	0.2	0.4	1.7	9.2
Acute and chronic pancreatitis	Partially attributable	0.1	0.0	0.4	0.9	2.0	2.3	5.0	14.0
Psoriasis	Partially attributable	0.0	0.0	0.0	0.0	0.0	0.0	0.1	0.0
Spontaneous abortion	Partially attributable	0.0	0.0	0.0	0.0	0.0	0.0	0.0	0.0
Road traffic accidents - non pediastrian	Partially attributable	13.6	15.3	11.4	7.3	5.8	3.8	3.4	7.4
Pedestrian traffic accidents	Partially attributable	2.1	1.6	1.2	1.2	0.9	1.1	1.4	4.7
Water transport accidents	Partially attributable	0.0	0.0	0.1	0.1	0.1	0.1	0.1	0.3
Air/space transport accidents	Partially attributable	0.1	0.0	0.1	0.2	0.1	0.3	0.0	0.0
Fall injuries	Partially attributable	0.6	1.1	1.0	2.0	3.9	5.8	11.0	54.6
Work/machine injuries	Partially attributable	0.1	0.2	0.2	0.2	0.1	0.1	0.1	0.1
Firearm injuries	Partially attributable	0.0	0.1	0.0	0.0	0.0	0.0	0.0	0.0
Drowning	Partially attributable	1.1	0.7	0.5	0.3	0.7	0.6	0.3	0.7
Inhalation of gastric contents	Partially attributable	0.0	0.0	0.2	0.3	0.4	0.7	0.9	2.7
Fire injuries	Partially attributable	0.0	0.0	0.0	0.0	0.0	0.0	0.0	0.0
Accidental excessive cold	Partially attributable	0.0	0.0	0.0	0.1	0.1	0.1	0.1	1.6
Intentional self-harm	Partially attributable	6.4	12.4	17.9	22.5	19.5	15.5	11.9	14.3
Assault	Partially attributable	0.8	1.9	1.4	1.2	0.9	0.6	0.4	0.4

Table A2: Female alcohol-related mortality rates (per 100,000) for England in 2005

Condition	Type	16-17	18-24	25-34	35-44	45-54	55-64	65-74	75+
Alcohol-induced pseudo-Cushing's syndrome	Wholly attributable	0.0	0.0	0.0	0.0	0.0	0.0	0.0	0.0
Mental and behavioural disorders due to use of alcohol	Wholly attributable	0.0	0.0	0.1	0.9	1.4	1.7	0.5	0.4
degeneration	Wholly attributable	0.0	0.0	0.0	0.0	0.0	0.0	0.0	0.0
Alcoholic polyneuropathy	Wholly attributable	0.0	0.0	0.0	0.0	0.0	0.0	0.0	0.0
Alcoholic myopathy	Wholly attributable	0.0	0.0	0.0	0.0	0.0	0.0	0.0	0.0
Alcoholic cardiomyopathy	Wholly attributable	0.0	0.0	0.0	0.0	0.1	0.1	0.0	0.0
Alcoholic gastritis	Wholly attributable	0.0	0.0	0.0	0.0	0.0	0.0	0.0	0.0
Alcoholic liver disease	Wholly attributable	0.0	0.1	1.3	6.6	13.1	13.6	8.8	3.0
Chronic pancreatitis	Wholly attributable	0.0	0.0	0.0	0.0	0.1	0.1	0.0	0.0
Ethanol poisoning	Wholly attributable	0.0	0.0	0.0	0.0	0.1	0.0	0.0	0.0
Methanol poisoning	Wholly attributable	0.0	0.0	0.0	0.0	0.0	0.0	0.0	0.0
Toxic effect of alcohol, unspecified	Wholly attributable	0.1	0.1	0.1	0.4	0.4	0.3	0.2	0.0
Accidental poisoning by exposure to alcohol	Wholly attributable	0.1	0.1	0.1	0.4	0.5	0.4	0.1	0.0
Malignant neoplasm of lip, oral cavity and pharynx	Partially attributable	0.1	0.0	0.1	0.6	1.6	3.3	5.2	11.4
Malignant neoplasm of oesophagus	Partially attributable	0.0	0.0	0.1	0.5	2.7	9.1	19.3	53.5
Malignant neoplasm of colon	Partially attributable	0.0	0.1	0.5	1.5	5.0	14.5	36.7	112.8
Malignant neoplasm of rectum	Partially attributable	0.0	0.1	0.1	0.3	2.1	5.1	12.9	34.5
Malignant neoplasm of liver and intrahepatic bile ducts	Partially attributable	0.0	0.0	0.1	0.2	1.5	4.3	11.3	22.4
Malignant neoplasm of larynx	Partially attributable	0.0	0.0	0.0	0.0	0.3	1.0	1.5	2.5
Malignant neoplasm of breast	Partially attributable	0.1	0.1	1.9	13.3	35.0	64.5	89.7	193.0
Diabetes mellitus (typeII)	Partially attributable	0.0	0.0	0.0	0.1	0.1	0.6	4.0	24.6
Epilepsy and status epilepticus	Partially attributable	1.0	1.4	1.2	1.1	1.7	1.6	2.2	5.4
Hypertensive diseases	Partially attributable	0.0	0.0	0.0	0.3	1.1	3.6	9.8	77.0
Ischaemic heart disease	Partially attributable	0.0	0.0	0.7	3.7	14.1	48.7	198.5	1151.0
Cardiac arrhythmias	Partially attributable	0.2	0.1	0.2	0.1	0.3	0.9	5.3	78.7
haemorrhagic stroke	Partially attributable	0.2	0.2	1.0	3.4	9.0	14.1	29.0	96.0
Ischaemic stroke	Partially attributable	0.1	0.1	0.3	0.6	2.8	9.2	61.7	659.4
Oesophageal varices	Partially attributable	0.0	0.0	0.0	0.0	0.0	0.1	0.0	0.3
Gastro-oesophageal laceration-haemorrhage syndrome	Partially attributable	0.0	0.0	0.0	0.0	0.0	0.1	0.0	0.4
Unspecified liver disease	Partially attributable	0.0	0.0	0.3	1.1	2.9	4.3	8.7	11.4
heart failure	Partially attributable	0.2	0.2	0.3	0.6	1.3	3.8	14.7	234.9
Cholelithiasis	Partially attributable	0.0	0.0	0.1	0.0	0.1	0.6	2.1	10.7
Acute and chronic pancreatitis	Partially attributable	0.1	0.0	0.1	0.5	0.9	1.3	3.0	13.8
Psoriasis	Partially attributable	0.0	0.0	0.0	0.0	0.0	0.0	0.0	0.1
Spontaneous abortion	Partially attributable	0.0	0.0	0.0	0.0	0.0	0.0	0.0	0.0
Road traffic accidents - non pediastrian	Partially attributable	4.4	3.8	1.8	1.2	1.5	1.4	1.6	3.0
Pedestrian traffic accidents	Partially attributable	0.8	0.7	0.6	0.2	0.3	0.4	0.8	3.5
Water transport accidents	Partially attributable	0.0	0.0	0.0	0.0	0.0	0.0	0.0	0.0
Air/space transport accidents	Partially attributable	0.0	0.0	0.0	0.0	0.0	0.1	0.0	0.0
Fall injuries	Partially attributable	0.0	0.1	0.2	0.7	1.7	2.4	6.1	51.6
Work/machine injuries	Partially attributable	0.1	0.1	0.0	0.0	0.0	0.0	0.0	0.0
Firearm injuries	Partially attributable	0.0	0.0	0.0	0.0	0.0	0.0	0.0	0.0
Drowning	Partially attributable	0.2	0.2	0.1	0.1	0.3	0.2	0.2	0.5
Inhalation of gastric contents	Partially attributable	0.0	0.0	0.0	0.1	0.3	0.3	0.3	2.6
Fire injuries	Partially attributable	0.0	0.0	0.0	0.0	0.0	0.0	0.0	0.0
Accidental excessive cold	Partially attributable	0.0	0.0	0.0	0.0	0.1	0.0	0.3	1.3
Intentional self-harm	Partially attributable	2.2	3.1	5.1	5.3	7.2	6.4	4.3	4.8
Assault	Partially attributable	0.5	0.6	0.4	0.7	0.4	0.2	0.2	0.2

Table A3: Male alcohol-related morbidity (person-specific hospital admission) rates (per 100,000) for England in 2005/6

Condition	Type	16-17	18-24	25-34	35-44	45-54	55-64	65-74	75+
Alcohol-induced pseudo-Cushing's syndrome	Wholly attributable	0.0	0.0	0.0	0.0	0.0	0.0	0.0	0.0
Mental and behavioural disorders due to use of alcohol	Wholly attributable	233.7	231.5	285.0	397.3	449.7	396.3	329.5	228.1
degeneration	Wholly attributable	0.0	0.0	0.0	0.8	2.2	2.2	2.1	1.6
Alcoholic polyneuropathy	Wholly attributable	0.0	0.0	0.2	0.2	0.8	1.1	1.0	0.7
Alcoholic myopathy	Wholly attributable	0.0	0.0	0.0	0.1	0.2	0.3	0.3	0.3
Alcoholic cardiomyopathy	Wholly attributable	0.0	0.0	0.2	1.2	3.7	5.4	5.2	2.4
Alcoholic gastritis	Wholly attributable	2.1	2.1	2.7	3.3	3.0	1.9	1.5	0.9
Alcoholic liver disease	Wholly attributable	0.6	0.6	6.9	31.7	71.7	88.4	74.1	34.5
Chronic pancreatitis	Wholly attributable	1.3	1.3	4.6	8.5	8.1	5.0	2.6	1.2
Ethanol poisoning	Wholly attributable	50.6	50.1	50.7	42.3	26.0	11.6	4.8	3.0
Methanol poisoning	Wholly attributable	0.0	0.0	0.1	0.1	0.1	0.0	0.0	0.1
Toxic effect of alcohol, unspecified	Wholly attributable	1.8	1.7	1.6	2.0	1.1	0.4	0.2	0.1
Accidental poisoning by exposure to alcohol	Wholly attributable	2.6	2.6	1.6	1.4	1.1	0.4	0.5	0.3
Malignant neoplasm of lip, oral cavity and pharynx	Partially attributable	0.7	0.7	1.6	5.4	23.0	38.2	45.2	46.4
Malignant neoplasm of oesophagus	Partially attributable	0.1	0.1	0.6	3.8	20.2	62.6	119.5	175.5
Malignant neoplasm of colon	Partially attributable	0.9	0.9	2.4	7.2	24.1	83.9	212.3	345.6
Malignant neoplasm of rectum	Partially attributable	0.2	0.2	0.9	3.9	20.0	62.7	136.9	167.2
Malignant neoplasm of liver and intrahepatic bile ducts	Partially attributable	0.5	0.5	0.5	1.3	3.7	10.2	23.3	36.3
Malignant neoplasm of larynx	Partially attributable	0.0	0.0	0.1	1.2	7.0	23.3	33.6	42.1
Malignant neoplasm of breast	Partially attributable	0.0	0.0	0.0	0.0	0.0	0.0	0.0	0.0
Diabetes mellitus (typeII)	Partially attributable	8.6	8.6	28.1	87.0	224.5	428.2	872.9	1174.0
Epilepsy and status epilepticus	Partially attributable	92.7	91.9	101.4	113.9	133.1	159.7	244.1	388.0
Hypertensive diseases	Partially attributable	19.5	19.3	88.2	335.9	1048.3	2375.3	4671.0	6241.6
Ischaemic heart disease	Partially attributable	3.7	3.6	21.7	122.7	410.6	869.5	1506.2	2152.7
Cardiac arrhythmias	Partially attributable	20.3	20.1	39.4	86.2	216.0	644.8	1848.3	4493.2
haemorrhagic stroke	Partially attributable	3.8	3.7	6.9	13.1	26.9	39.6	74.1	145.2
Ischaemic stroke	Partially attributable	1.7	1.7	4.9	15.7	52.5	151.8	387.4	818.4
Oesophageal varices	Partially attributable	1.6	1.6	2.1	4.5	11.4	15.5	19.8	18.2
Gastro-oesophageal laceration-haemorrhage syndrome	Partially attributable	10.0	10.0	9.3	6.7	4.1	6.0	12.1	23.3
Unspecified liver disease	Partially attributable	2.3	2.3	4.2	15.7	24.2	30.1	34.1	43.5
heart failure	Partially attributable	5.7	5.7	7.4	10.5	19.6	39.6	96.7	328.0
Cholelithiasis	Partially attributable	7.0	6.9	27.0	58.5	101.2	164.8	330.2	544.6
Acute and chronic pancreatitis	Partially attributable	7.6	7.5	17.6	30.4	41.8	52.9	74.6	107.4
Psoriasis	Partially attributable	4.6	4.6	8.9	13.3	15.9	17.7	16.5	16.3
Spontaneous abortion	Partially attributable	0.0	0.0	0.1	0.1	0.0	0.0	0.0	0.0
Road traffic accidents - non pediastrian	Partially attributable	164.8	163.2	92.4	75.0	53.7	41.6	29.7	42.2
Pedestrian traffic accidents	Partially attributable	3.1	3.0	2.5	2.0	1.6	1.3	1.4	3.8
Water transport accidents	Partially attributable	1.0	1.0	1.4	1.4	0.8	1.1	0.6	0.5
Air/space transport accidents	Partially attributable	0.2	0.2	0.5	0.8	0.6	0.3	0.4	0.0
Fall injuries	Partially attributable	285.5	282.8	226.3	195.7	207.2	273.7	555.0	2545.5
Work/machine injuries	Partially attributable	151.4	150.0	127.9	95.7	69.6	52.9	32.3	18.5
Firearm injuries	Partially attributable	9.2	9.1	3.4	2.0	1.5	0.9	0.6	0.3
Drowning	Partially attributable	0.4	0.4	0.1	0.3	0.3	0.4	0.4	0.7
Inhalation of gastric contents	Partially attributable	1.1	1.1	1.2	1.5	1.9	2.9	7.5	21.3
Fire injuries	Partially attributable	9.8	9.7	7.2	5.2	4.3	3.6	4.6	7.5
Accidental excessive cold	Partially attributable	0.2	0.2	0.1	0.2	0.2	0.4	0.7	3.7
Intentional self-harm	Partially attributable	156.3	154.9	139.9	108.7	67.1	37.6	26.9	42.1
Assault	Partially attributable	364.4	360.9	210.6	122.4	61.2	23.4	12.0	9.6

Table A4: Female alcohol-related morbidity (person-specific hospital admission) rates (per 100,000) for England in 2005/6

Condition	Type	16-17	18-24	25-34	35-44	45-54	55-64	65-74	75+
Alcohol-induced pseudo-Cushing's syndrome	Wholly attributable	0.0	0.0	0.0	0.0	0.0	0.0	0.0	0.0
Mental and behavioural disorders due to use of alcohol	Wholly attributable	112.3	109.7	107.1	155.1	177.1	126.2	90.0	67.9
degeneration	Wholly attributable	0.0	0.0	0.0	0.4	0.9	0.9	0.5	0.4
Alcoholic polyneuropathy	Wholly attributable	0.0	0.0	0.1	0.1	0.5	0.3	0.2	0.2
Alcoholic myopathy	Wholly attributable	0.0	0.0	0.0	0.0	0.0	0.0	0.0	0.0
Alcoholic cardiomyopathy	Wholly attributable	0.0	0.0	0.0	0.2	0.3	0.4	0.1	0.4
Alcoholic gastritis	Wholly attributable	1.0	1.0	0.6	1.0	0.9	0.6	0.4	0.1
Alcoholic liver disease	Wholly attributable	0.3	0.3	5.0	17.2	34.7	36.0	30.0	11.1
Chronic pancreatitis	Wholly attributable	0.4	0.4	1.0	2.0	2.2	1.0	0.8	0.1
Ethanol poisoning	Wholly attributable	85.0	82.9	58.4	59.2	37.7	12.7	4.8	1.8
Methanol poisoning	Wholly attributable	0.1	0.1	0.0	0.1	0.1	0.0	0.0	0.1
Toxic effect of alcohol, unspecified	Wholly attributable	2.8	2.7	1.9	1.9	1.2	0.7	0.2	0.0
Accidental poisoning by exposure to alcohol	Wholly attributable	2.7	2.6	1.4	1.4	0.9	0.4	0.1	0.3
Malignant neoplasm of lip, oral cavity and pharynx	Partially attributable	0.7	0.7	1.6	3.5	9.8	16.4	22.6	24.9
Malignant neoplasm of oesophagus	Partially attributable	0.1	0.1	0.1	1.6	6.6	19.9	42.0	80.2
Malignant neoplasm of colon	Partially attributable	0.6	0.6	2.1	6.9	22.9	62.9	144.4	226.2
Malignant neoplasm of rectum	Partially attributable	0.3	0.3	1.2	3.6	11.7	29.3	59.1	78.9
Malignant neoplasm of liver and intrahepatic bile ducts	Partially attributable	0.3	0.3	0.4	0.8	2.6	6.0	13.5	20.4
Malignant neoplasm of larynx	Partially attributable	0.0	0.0	0.1	0.4	1.9	4.1	5.7	6.7
Malignant neoplasm of breast	Partially attributable	1.1	1.1	24.6	127.3	273.3	340.0	323.8	219.8
Diabetes mellitus (typeII)	Partially attributable	10.6	10.3	32.8	83.9	179.2	290.6	570.1	746.9
Epilepsy and status epilepticus	Partially attributable	126.1	123.2	138.9	128.8	138.4	153.7	199.7	294.3
Hypertensive diseases	Partially attributable	25.2	24.6	93.0	318.2	979.0	2154.7	4400.5	6122.4
Ischaemic heart disease	Partially attributable	2.1	2.0	7.9	44.5	162.4	356.6	733.5	1244.7
Cardiac arrhythmias	Partially attributable	25.3	24.7	38.0	51.6	111.6	327.5	1144.8	3625.2
haemorrhagic stroke	Partially attributable	3.3	3.2	5.5	13.8	26.5	34.6	59.6	123.0
Ischaemic stroke	Partially attributable	2.1	2.1	6.0	12.9	32.6	78.7	246.8	725.5
Oesophageal varices	Partially attributable	1.2	1.2	1.5	2.4	5.4	9.5	15.0	11.1
Gastro-oesophageal laceration-haemorrhage syndrome	Partially attributable	8.8	8.6	5.1	3.2	2.8	3.9	7.0	13.7
Unspecified liver disease	Partially attributable	2.6	2.5	3.9	8.3	17.3	32.6	57.2	48.5
heart failure	Partially attributable	4.1	4.0	6.0	7.1	13.1	28.3	75.4	300.9
Cholelithiasis	Partially attributable	100.3	98.0	201.9	236.3	277.5	356.8	442.3	562.7
Acute and chronic pancreatitis	Partially attributable	17.2	16.8	24.2	27.7	35.8	49.3	69.4	93.0
Psoriasis	Partially attributable	10.9	10.7	13.2	11.2	12.1	14.4	13.4	13.3
Spontaneous abortion	Partially attributable	329.0	321.2	511.8	280.2	10.0	0.1	0.0	3.9
Road traffic accidents - non pediastrian	Partially attributable	65.6	64.0	40.2	24.8	22.2	21.4	23.1	30.5
Pedestrian traffic accidents	Partially attributable	1.8	1.8	1.1	0.9	1.0	1.8	1.6	3.2
Water transport accidents	Partially attributable	0.3	0.3	0.3	0.4	0.4	0.4	0.5	0.5
Air/space transport accidents	Partially attributable	0.0	0.0	0.2	0.1	0.1	0.1	0.0	0.0
Fall injuries	Partially attributable	147.5	144.0	151.1	140.9	200.9	373.2	896.9	4610.6
Work/machine injuries	Partially attributable	39.1	38.2	30.4	24.0	17.6	11.1	8.4	6.7
Firearm injuries	Partially attributable	0.9	0.9	0.3	0.4	0.1	0.2	0.0	0.0
Drowning	Partially attributable	0.3	0.3	0.1	0.2	0.1	0.2	0.2	0.5
Inhalation of gastric contents	Partially attributable	0.7	0.6	1.0	1.2	1.5	2.4	4.2	16.3
Fire injuries	Partially attributable	2.8	2.7	2.5	2.2	1.9	2.0	2.7	6.9
Accidental excessive cold	Partially attributable	0.0	0.0	0.1	0.1	0.1	0.1	0.6	4.4
Intentional self-harm	Partially attributable	323.3	315.7	169.6	146.2	96.5	48.4	34.6	37.4
Assault	Partially attributable	61.7	60.3	41.1	25.4	12.6	5.1	4.4	9.2

1.1.2. Step 2: Derivation of age, gender and SEC-specific adjustment factors for alcohol-related mortality rates.

Siegler et al. derived age, gender and SEC group-specific alcohol-related mortality rates for England and Wales using 2001-2003 ONS data (reproduced in Tables A5-A6).[3] These are the most recent data of this kind available. As the age groups defined by Siegler et al. are different to those used in SAPM, ONS mid-2010 population estimates are used as weights to allow estimation of rates for the modelled age groups (Tables A7-A8).[4] Next, the alcohol-related mortality rate for each SEC group within an age and sex group is expressed as a ratio of the total alcohol-related mortality rate for that age and sex group (Tables A9-A10).

Table A5: Male alcohol-related mortality rates (per 100,000) by age and SEC classification, England and Wales, 2001-2003

	25-29	30-34	35-39	40-44	45-49	50-54	55-59	60-64
**NS-SEC1.1**	n/a	0.7	1.3	2.6	7.4	13.8	14.2	17.6
**NS-SEC1.2**	n/a	2.0	4.3	8.5	12.4	20.9	20.9	27.6
**NS-SEC2**	0.3	1.2	5.1	13.3	19.4	25.6	30.3	28.4
**NS-SEC3**	0.9	3.9	6.1	11.1	26.4	34.4	30.2	26.1
**NS-SEC4**	1.8	6.5	8.1	15.3	25.9	27.8	27.8	29.0
**NS-SEC5**	1.7	3.6	8.6	18.6	27.9	37.9	39.1	34.3
**NS-SEC6**	2.8	8.6	16.9	34.5	46.6	42.5	48.9	42.0
**NS-SEC7**	3.8	10.0	20.4	35.7	49.1	52.2	45.3	41.1
**All**	1.5	4.4	9.0	18.1	27.1	32.1	33.2	32.5

Source: Table 6 in Siegler et al.[3]

Table A6: Female alcohol-related mortality rates (per 100,000) by age and SEC classification, England and Wales, 2001-2003

	25-29	30-34	35-39	40-44	45-49	50-54	55-59	60-64
**NS-SEC1.1**	n/a	1.0	1.8	3.2	7.3	7.4	7.4	n/a
**NS-SEC1.2**	0.7	n/a	2.2	3.1	5.6	6.9	8.7	0.7
**NS-SEC2**	0.2	1.2	2.8	7.0	9.7	12.8	12.3	0.2
**NS-SEC3**	0.7	2.2	5.5	9.5	13.3	14.5	12.2	0.7
**NS-SEC4**	n/a	1.1	4.1	8.6	13.6	14.2	17.1	n/a
**NS-SEC5**	2.0	2.7	7.1	11.3	18.5	22.5	24.2	2.0
**NS-SEC6**	2.7	5.2	7.5	15.8	21.7	22.1	20.1	2.7
**NS-SEC7**	1.0	6.2	13.6	27.4	42.0	38.2	29.5	1.0
**All**	1.0	2.3	4.9	9.6	14.3	16.1	16.0	1.0

Source: Table 7 in Siegler et al.[3]

Table A7: Males alcohol-related mortality rates (per 100,000) by SAPM age groups and SEC classification, England and Wales, 2001-2003

	25-34	35-44	45-54	55-64
**NS-SEC1.1**	0.7	2.0	10.4	15.9
**NS-SEC1.2**	2.0	6.5	16.4	24.3
**NS-SEC2**	0.7	9.3	22.3	29.3
**NS-SEC3**	2.4	8.7	30.1	28.1
**NS-SEC4**	4.1	11.8	26.8	28.4
**NS-SEC5**	2.6	13.8	32.6	36.6
**NS-SEC6**	5.6	26.0	44.7	45.4
**NS-SEC7**	6.8	28.3	50.5	43.1
**All**	2.9	13.7	29.4	32.8

Table A8: Female alcohol-related mortality rates (per 100,000) by SAPM age groups and SEC classification, England and Wales, 2001-2003

	25-34	35-44	45-54	55-64
**NS-SEC1.1**	1.0	2.5	7.3	7.4
**NS-SEC1.2**	0.7	2.7	6.2	8.7
**NS-SEC2**	0.7	5.0	11.1	12.3
**NS-SEC3**	1.4	7.6	13.9	12.2
**NS-SEC4**	1.1	6.4	13.9	17.1
**NS-SEC5**	2.3	9.3	20.4	24.2
**NS-SEC6**	3.9	11.8	21.9	20.1
**NS-SEC7**	3.5	20.8	40.2	29.5
**All**	1.6	7.3	15.1	16.0

Table A9: Male ratios of the mortality rate of each age-sex-SEC group to the mortality rate of the age-sex group, England and Wales, 2001-2003

	25-34	35-44	45-54	55-64
**NS-SEC1.1**	0.24	0.14	0.35	0.49
**NS-SEC1.2**	0.69	0.47	0.56	0.74
**NS-SEC2**	0.25	0.68	0.76	0.89
**NS-SEC3**	0.81	0.63	1.02	0.86
**NS-SEC4**	1.40	0.86	0.91	0.87
**NS-SEC5**	0.90	1.00	1.11	1.12
**NS-SEC6**	1.93	1.90	1.52	1.38
**NS-SEC7**	2.34	2.07	1.72	1.31

Table A10: Female ratios of the mortality rate of each age-sex-SEC group to the mortality rate of the age-sex group, England and Wales, 2001-2003

	25-34	35-44	45-54	55-64
**NS-SEC1.1**	0.61	0.34	0.49	0.46
**NS-SEC1.2**	0.43	0.36	0.41	0.54
**NS-SEC2**	0.42	0.68	0.74	0.77
**NS-SEC3**	0.88	1.03	0.92	0.76
**NS-SEC4**	0.67	0.88	0.92	1.07
**NS-SEC5**	1.43	1.26	1.34	1.51
**NS-SEC6**	2.40	1.61	1.45	1.26
**NS-SEC7**	2.16	2.83	2.66	1.84

1.1.3. Step 3: Estimation of SEC-specific alcohol-related mortality rates using SAPM.

SAPM already implicitly accounts for differences in drinking patterns between SEC groups; therefore, an assessment is required of the extent to which the differences in alcohol-related mortality between SEC groups seen in Tables A9-A10 are solely due to drinking patterns. This assessment is undertaken by modelling within SAPM a scenario where everybody stops drinking and using the resulting estimated harm reductions to derive alcohol-related mortality rates for each age, gender and SEC group. If the estimated alcohol-related mortality rates are not comparable to the patterns in Tables A5-A10, then this indicates the differential mortality rates are not solely attributable to differences in drinking patterns across SEC groups. Adjustments to the underlying absolute alcohol-related mortality risks used in SAPM would then be required.

Table A11 presents the estimated alcohol-related deaths and mortality rates under the ‘everybody stops drinking scenario’. It also presents ratios of the alcohol-related mortality rate for each SEC group to the population alcohol-related mortality rate. Unlike Tables A5-A10, the results from SAPM show higher alcohol-related mortality rates for higher SEC groups. Although contrary to the evidence from Siegler et al., this is expected as higher SEC groups drink more on average than lower SEC groups and, therefore, SAPM would be expected to estimate a higher alcohol-related mortality rate for higher SEC groups. Adjustments to SAPM to account for this are required.

Table A11: SAPM-derived estimated alcohol-related deaths and mortality rates and ratios of the alcohol-attributable mortality rate of each SEC group to the alcohol-attributable rate of the population

	Alcohol-attributable deaths	Alcohol-attributable mortality rate (per 100,000)	Ratios of NS-SEC rate to total rate
**NS-SEC1.1**	859.0	29.2	1.32
**NS-SEC1.2**	1187.1	28.7	1.30
**NS-SEC2**	2737.3	24.3	1.10
**NS-SEC3**	608.3	16.7	0.75
**NS-SEC4**	863.1	21.1	0.95
**NS-SEC5**	899.9	18.3	0.83
**NS-SEC6**	920.3	16.6	0.75
**NS-SEC7**	1004.0	22.6	1.02
**Total**	9079.0	22.2	1.00

1.1.4. Step 4: Modifying adjustment factors to account for SEC-related differences in drinking patterns already modelled in SAPM.

Adjustment factors are calculated by dividing the estimated age, gender and SEC group ratios in *Step 2* by the corresponding SEC group ratio in *Step 3*. For example, Table A9 gives a ratio of 0.24 for 25-34 year-old men in NS-SEC Group 1.1 based on evidence from Siegler et al.[3] and the corresponding ratio of 1.32 from is estimated for NS-SEC Group 1.1 in SAPM. Therefore, the final adjustment factor for 25-34 year-old men in NS-SEC Group 1.1 is estimated to be 0.18 (0.24 / 1.32 = 0.18). The adjustment factors are shown in Tables A12-A13. These are applied to all of the conditions in Tables A1-A2 to derive age, gender and SEC group-specific mortality rates. For example, the original mortality rate for alcoholic liver disease for 25-34 year-old men is 2.6 per 100,000. Applying the adjustment factors gives a mortality rate of 0.5 per 100,000 for 25-34 year-old men in NS-SEC group 1.1 (2.6 * 0.18 = 0.5) and 6.0 per 100,000 for counterparts in NS-SEC group 7 (2.6 * 2.30 = 6.0).

Given the data reported by Siegler et al., it is not possible to derive adjustment factors for the following age groups modelled in SAPM: 16-17, 18-24, 65-74 and 75+. Therefore, it is assumed that the adjustment factors for the younger age groups are the same as for 25-34 year-olds and for the older age groups are the same as 55-64 year-olds. It is also not possible to derive adjustment factors from Siegler et al. for NS-SEC Group 8 (never worked and long-term unemployed); therefore, it is assumed that NS-SEC Group 8 has the same mortality rates as the general population (i.e. they are assigned the mortality rates shown in Tables A1-A2). A sensitivity analysis (reported below) tests the assumption that NS-SEC Group 8 has the same mortality rate as NS-SEC Group 7. The adjusted mortality rates for NS-SEC Group 1.1 are presented in Tables A14-15. Results for other NS-SEC groups are available on request.

Table A12: Male adjustment factors for alcohol-related mortality rates by NS-SEC group

	25-34	35-44	45-54	55-64
**NS-SEC1.1**	0.18	0.11	0.27	0.37
**NS-SEC1.2**	0.53	0.36	0.43	0.57
**NS-SEC2**	0.23	0.62	0.69	0.81
**NS-SEC3**	1.08	0.84	1.36	1.14
**NS-SEC4**	1.48	0.91	0.96	0.91
**NS-SEC5**	1.09	1.22	1.34	1.35
**NS-SEC6**	2.58	2.53	2.03	1.84
**NS-SEC7**	2.30	2.03	1.68	1.29

Table A13: Female adjustment factors for alcohol-related mortality rates by NS-SEC group

	25-34	35-44	45-54	55-64
**NS-SEC1.1**	0.47	0.26	0.37	0.35
**NS-SEC1.2**	0.33	0.28	0.32	0.42
**NS-SEC2**	0.38	0.62	0.67	0.70
**NS-SEC3**	1.16	1.37	1.22	1.01
**NS-SEC4**	0.71	0.92	0.96	1.12
**NS-SEC5**	1.74	1.53	1.63	1.83
**NS-SEC6**	3.20	2.15	1.93	1.68
**NS-SEC7**	2.12	2.78	2.61	1.81

Table A14: Male SEC-adjusted alcohol-related mortality rates (per 100,000) for England in 2005 for SEC1.1

Condition	Type	16-17	18-24	25-34	35-44	45-54	55-64	65-74	75+
Alcohol-induced pseudo-Cushing's syndrome	Wholly attributable	0.0	0.0	0.0	0.0	0.0	0.0	0.0	0.0
Mental and behavioural disorders due to use of alcohol	Wholly attributable	0.0	0.0	0.1	0.2	0.7	1.2	0.9	0.5
degeneration	Wholly attributable	0.0	0.0	0.0	0.0	0.0	0.0	0.0	0.1
Alcoholic polyneuropathy	Wholly attributable	0.0	0.0	0.0	0.0	0.0	0.0	0.0	0.0
Alcoholic myopathy	Wholly attributable	0.0	0.0	0.0	0.0	0.0	0.0	0.0	0.0
Alcoholic cardiomyopathy	Wholly attributable	0.0	0.0	0.0	0.0	0.2	0.3	0.3	0.0
Alcoholic gastritis	Wholly attributable	0.0	0.0	0.0	0.0	0.0	0.0	0.0	0.0
Alcoholic liver disease	Wholly attributable	0.0	0.0	0.5	1.3	7.2	10.4	7.5	3.1
Chronic pancreatitis	Wholly attributable	0.0	0.0	0.0	0.0	0.1	0.1	0.1	0.0
Ethanol poisoning	Wholly attributable	0.0	0.0	0.0	0.0	0.0	0.0	0.0	0.0
Methanol poisoning	Wholly attributable	0.0	0.0	0.0	0.0	0.0	0.0	0.0	0.0
Toxic effect of alcohol, unspecified	Wholly attributable	0.0	0.0	0.1	0.1	0.3	0.2	0.1	0.0
Accidental poisoning by exposure to alcohol	Wholly attributable	0.0	0.0	0.1	0.1	0.3	0.2	0.1	0.0
Malignant neoplasm of lip, oral cavity and pharynx	Partially attributable	0.0	0.0	0.1	0.1	1.1	3.7	5.3	6.5
Malignant neoplasm of oesophagus	Partially attributable	0.0	0.0	0.0	0.1	2.4	10.2	21.8	40.9
Malignant neoplasm of colon	Partially attributable	0.0	0.0	0.1	0.1	1.5	7.1	21.2	54.9
Malignant neoplasm of rectum	Partially attributable	0.0	0.0	0.0	0.1	1.0	4.4	11.2	23.6
Malignant neoplasm of liver and intrahepatic bile ducts	Partially attributable	0.0	0.0	0.1	0.1	1.0	3.3	8.5	15.7
Malignant neoplasm of larynx	Partially attributable	0.0	0.0	0.0	0.0	0.3	1.5	2.2	5.2
Malignant neoplasm of breast	Partially attributable	0.0	0.0	0.0	0.0	0.0	0.2	0.3	0.8
Diabetes mellitus (typeII)	Partially attributable	0.0	0.0	0.0	0.0	0.1	0.4	2.2	9.0
Epilepsy and status epilepticus	Partially attributable	0.2	0.2	0.3	0.3	0.8	0.9	1.0	2.1
Hypertensive diseases	Partially attributable	0.0	0.0	0.0	0.1	0.8	2.5	5.7	22.3
Ischaemic heart disease	Partially attributable	0.0	0.0	0.5	1.9	18.9	69.8	180.8	613.5
Cardiac arrhythmias	Partially attributable	0.0	0.0	0.0	0.0	0.1	0.3	2.0	17.3
haemorrhagic stroke	Partially attributable	0.1	0.1	0.2	0.4	2.6	5.5	11.3	33.5
Ischaemic stroke	Partially attributable	0.0	0.0	0.1	0.1	1.2	6.6	30.7	202.5
Oesophageal varices	Partially attributable	0.0	0.0	0.0	0.0	0.0	0.1	0.1	0.2
Gastro-oesophageal laceration-haemorrhage syndrome	Partially attributable	0.0	0.0	0.0	0.0	0.0	0.1	0.0	0.2
Unspecified liver disease	Partially attributable	0.0	0.0	0.0	0.2	1.7	3.1	4.0	4.8
heart failure	Partially attributable	0.1	0.1	0.1	0.1	0.7	2.2	7.9	72.8
Cholelithiasis	Partially attributable	0.0	0.0	0.0	0.0	0.0	0.1	0.6	3.4
Acute and chronic pancreatitis	Partially attributable	0.0	0.0	0.1	0.1	0.5	0.9	1.8	5.1
Psoriasis	Partially attributable	0.0	0.0	0.0	0.0	0.0	0.0	0.0	0.0
Spontaneous abortion	Partially attributable	0.0	0.0	0.0	0.0	0.0	0.0	0.0	0.0
Road traffic accidents - non pediastrian	Partially attributable	2.5	2.8	2.1	0.8	1.5	1.4	1.2	2.7
Pedestrian traffic accidents	Partially attributable	0.4	0.3	0.2	0.1	0.2	0.4	0.5	1.7
Water transport accidents	Partially attributable	0.0	0.0	0.0	0.0	0.0	0.0	0.1	0.1
Air/space transport accidents	Partially attributable	0.0	0.0	0.0	0.0	0.0	0.1	0.0	0.0
Fall injuries	Partially attributable	0.1	0.2	0.2	0.2	1.0	2.1	4.0	20.1
Work/machine injuries	Partially attributable	0.0	0.0	0.0	0.0	0.0	0.0	0.1	0.0
Firearm injuries	Partially attributable	0.0	0.0	0.0	0.0	0.0	0.0	0.0	0.0
Drowning	Partially attributable	0.2	0.1	0.1	0.0	0.2	0.2	0.1	0.3
Inhalation of gastric contents	Partially attributable	0.0	0.0	0.0	0.0	0.1	0.2	0.3	1.0
Fire injuries	Partially attributable	0.0	0.0	0.0	0.0	0.0	0.0	0.0	0.0
Accidental excessive cold	Partially attributable	0.0	0.0	0.0	0.0	0.0	0.0	0.1	0.6
Intentional self-harm	Partially attributable	1.2	2.3	3.3	2.5	5.2	5.7	4.4	5.3
Assault	Partially attributable	0.1	0.3	0.3	0.1	0.2	0.2	0.1	0.2

Table A15: Female SEC-adjusted alcohol-related mortality rates (per 100,000) for England in 2005 for SEC1.1

Condition	Type	16-17	18-24	25-34	35-44	45-54	55-64	65-74	75+
Alcohol-induced pseudo-Cushing's syndrome	Wholly attributable	0.0	0.0	0.0	0.0	0.0	0.0	0.0	0.0
Mental and behavioural disorders due to use of alcohol	Wholly attributable	0.0	0.0	0.1	0.2	0.5	0.6	0.2	0.1
degeneration	Wholly attributable	0.0	0.0	0.0	0.0	0.0	0.0	0.0	0.0
Alcoholic polyneuropathy	Wholly attributable	0.0	0.0	0.0	0.0	0.0	0.0	0.0	0.0
Alcoholic myopathy	Wholly attributable	0.0	0.0	0.0	0.0	0.0	0.0	0.0	0.0
Alcoholic cardiomyopathy	Wholly attributable	0.0	0.0	0.0	0.0	0.0	0.0	0.0	0.0
Alcoholic gastritis	Wholly attributable	0.0	0.0	0.0	0.0	0.0	0.0	0.0	0.0
Alcoholic liver disease	Wholly attributable	0.0	0.0	0.6	1.7	4.8	4.8	3.1	1.1
Chronic pancreatitis	Wholly attributable	0.0	0.0	0.0	0.0	0.0	0.0	0.0	0.0
Ethanol poisoning	Wholly attributable	0.0	0.0	0.0	0.0	0.0	0.0	0.0	0.0
Methanol poisoning	Wholly attributable	0.0	0.0	0.0	0.0	0.0	0.0	0.0	0.0
Toxic effect of alcohol, unspecified	Wholly attributable	0.0	0.0	0.0	0.1	0.1	0.1	0.1	0.0
Accidental poisoning by exposure to alcohol	Wholly attributable	0.0	0.0	0.1	0.1	0.2	0.1	0.0	0.0
Malignant neoplasm of lip, oral cavity and pharynx	Partially attributable	0.0	0.0	0.1	0.2	0.6	1.2	1.8	4.0
Malignant neoplasm of oesophagus	Partially attributable	0.0	0.0	0.0	0.1	1.0	3.2	6.8	18.8
Malignant neoplasm of colon	Partially attributable	0.0	0.0	0.2	0.4	1.9	5.1	12.9	39.6
Malignant neoplasm of rectum	Partially attributable	0.0	0.0	0.1	0.1	0.8	1.8	4.5	12.1
Malignant neoplasm of liver and intrahepatic bile ducts	Partially attributable	0.0	0.0	0.1	0.1	0.5	1.5	4.0	7.9
Malignant neoplasm of larynx	Partially attributable	0.0	0.0	0.0	0.0	0.1	0.3	0.5	0.9
Malignant neoplasm of breast	Partially attributable	0.0	0.0	0.9	3.5	12.9	22.6	31.5	67.7
Diabetes mellitus (typeII)	Partially attributable	0.0	0.0	0.0	0.0	0.0	0.2	1.4	8.6
Epilepsy and status epilepticus	Partially attributable	0.5	0.6	0.5	0.3	0.6	0.6	0.8	1.9
Hypertensive diseases	Partially attributable	0.0	0.0	0.0	0.1	0.4	1.3	3.4	27.0
Ischaemic heart disease	Partially attributable	0.0	0.0	0.3	1.0	5.2	17.1	69.6	403.8
Cardiac arrhythmias	Partially attributable	0.1	0.0	0.1	0.0	0.1	0.3	1.9	27.6
haemorrhagic stroke	Partially attributable	0.1	0.1	0.5	0.9	3.3	5.0	10.2	33.7
Ischaemic stroke	Partially attributable	0.0	0.0	0.1	0.2	1.0	3.2	21.6	231.3
Oesophageal varices	Partially attributable	0.0	0.0	0.0	0.0	0.0	0.0	0.0	0.1
Gastro-oesophageal laceration-haemorrhage syndrome	Partially attributable	0.0	0.0	0.0	0.0	0.0	0.0	0.0	0.1
Unspecified liver disease	Partially attributable	0.0	0.0	0.1	0.3	1.1	1.5	3.1	4.0
heart failure	Partially attributable	0.1	0.1	0.1	0.1	0.5	1.3	5.2	82.4
Cholelithiasis	Partially attributable	0.0	0.0	0.0	0.0	0.0	0.2	0.8	3.7
Acute and chronic pancreatitis	Partially attributable	0.0	0.0	0.1	0.1	0.3	0.5	1.0	4.8
Psoriasis	Partially attributable	0.0	0.0	0.0	0.0	0.0	0.0	0.0	0.0
Spontaneous abortion	Partially attributable	0.0	0.0	0.0	0.0	0.0	0.0	0.0	0.0
Road traffic accidents - non pediastrian	Partially attributable	2.0	1.7	0.8	0.3	0.5	0.5	0.6	1.1
Pedestrian traffic accidents	Partially attributable	0.4	0.3	0.3	0.1	0.1	0.1	0.3	1.2
Water transport accidents	Partially attributable	0.0	0.0	0.0	0.0	0.0	0.0	0.0	0.0
Air/space transport accidents	Partially attributable	0.0	0.0	0.0	0.0	0.0	0.0	0.0	0.0
Fall injuries	Partially attributable	0.0	0.0	0.1	0.2	0.6	0.8	2.1	18.1
Work/machine injuries	Partially attributable	0.0	0.0	0.0	0.0	0.0	0.0	0.0	0.0
Firearm injuries	Partially attributable	0.0	0.0	0.0	0.0	0.0	0.0	0.0	0.0
Drowning	Partially attributable	0.1	0.1	0.0	0.0	0.1	0.1	0.1	0.2
Inhalation of gastric contents	Partially attributable	0.0	0.0	0.0	0.0	0.1	0.1	0.1	0.9
Fire injuries	Partially attributable	0.0	0.0	0.0	0.0	0.0	0.0	0.0	0.0
Accidental excessive cold	Partially attributable	0.0	0.0	0.0	0.0	0.0	0.0	0.1	0.5
Intentional self-harm	Partially attributable	1.0	1.5	2.3	1.4	2.7	2.3	1.5	1.7
Assault	Partially attributable	0.2	0.3	0.2	0.2	0.1	0.1	0.1	0.1

1.1.5. Step 5: Applying mortality adjustment factors to morbidity data.

As Siegler et al. only examined alcohol-related mortality and no comparable evidence is available relating to alcohol-related morbidity, the mortality adjustment factors in Tables A12-A13 and the method described above are used to adjust alcohol-related morbidity rates for the different SEC groups. The adjusted morbidity rates for NS-SEC group 1.1 are presented in Tables A16-17. Results for other NS-SEC groups are available on request.

Table A16: Male SEC-adjusted alcohol-related morbidity rates (per 100,000) for England in 2005 for SEC1.1

Condition	Type	16-17	18-24	25-34	35-44	45-54	55-64	65-74	75+
Alcohol-induced pseudo-Cushing's syndrome	Wholly attributable	0.0	0.0	0.0	0.0	0.0	0.0	0.0	0.0
Mental and behavioural disorders due to use of alcohol	Wholly attributable	42.5	42.1	51.9	43.4	120.3	145.9	121.3	84.0
degeneration	Wholly attributable	0.0	0.0	0.0	0.1	0.6	0.8	0.8	0.6
Alcoholic polyneuropathy	Wholly attributable	0.0	0.0	0.0	0.0	0.2	0.4	0.4	0.3
Alcoholic myopathy	Wholly attributable	0.0	0.0	0.0	0.0	0.1	0.1	0.1	0.1
Alcoholic cardiomyopathy	Wholly attributable	0.0	0.0	0.0	0.1	1.0	2.0	1.9	0.9
Alcoholic gastritis	Wholly attributable	0.4	0.4	0.5	0.4	0.8	0.7	0.6	0.3
Alcoholic liver disease	Wholly attributable	0.1	0.1	1.3	3.5	19.2	32.6	27.3	12.7
Chronic pancreatitis	Wholly attributable	0.2	0.2	0.8	0.9	2.2	1.8	1.0	0.4
Ethanol poisoning	Wholly attributable	9.2	9.1	9.2	4.6	6.9	4.3	1.8	1.1
Methanol poisoning	Wholly attributable	0.0	0.0	0.0	0.0	0.0	0.0	0.0	0.0
Toxic effect of alcohol, unspecified	Wholly attributable	0.3	0.3	0.3	0.2	0.3	0.2	0.1	0.0
Accidental poisoning by exposure to alcohol	Wholly attributable	0.5	0.5	0.3	0.1	0.3	0.2	0.2	0.1
Malignant neoplasm of lip, oral cavity and pharynx	Partially attributable	0.1	0.1	0.3	0.6	6.1	14.1	16.7	17.1
Malignant neoplasm of oesophagus	Partially attributable	0.0	0.0	0.1	0.4	5.4	23.0	44.0	64.6
Malignant neoplasm of colon	Partially attributable	0.2	0.2	0.4	0.8	6.5	30.9	78.2	127.2
Malignant neoplasm of rectum	Partially attributable	0.0	0.0	0.2	0.4	5.3	23.1	50.4	61.6
Malignant neoplasm of liver and intrahepatic bile ducts	Partially attributable	0.1	0.1	0.1	0.1	1.0	3.7	8.6	13.4
Malignant neoplasm of larynx	Partially attributable	0.0	0.0	0.0	0.1	1.9	8.6	12.4	15.5
Malignant neoplasm of breast	Partially attributable	0.0	0.0	0.0	0.0	0.0	0.0	0.0	0.0
Diabetes mellitus (typeII)	Partially attributable	1.6	1.6	5.1	9.5	60.1	157.6	321.4	432.2
Epilepsy and status epilepticus	Partially attributable	16.9	16.7	18.5	12.4	35.6	58.8	89.9	142.9
Hypertensive diseases	Partially attributable	3.5	3.5	16.1	36.7	280.4	874.5	1719.8	2298.0
Ischaemic heart disease	Partially attributable	0.7	0.7	3.9	13.4	109.8	320.1	554.6	792.6
Cardiac arrhythmias	Partially attributable	3.7	3.7	7.2	9.4	57.8	237.4	680.5	1654.3
haemorrhagic stroke	Partially attributable	0.7	0.7	1.3	1.4	7.2	14.6	27.3	53.4
Ischaemic stroke	Partially attributable	0.3	0.3	0.9	1.7	14.1	55.9	142.6	301.3
Oesophageal varices	Partially attributable	0.3	0.3	0.4	0.5	3.1	5.7	7.3	6.7
Gastro-oesophageal laceration-haemorrhage syndrome	Partially attributable	1.8	1.8	1.7	0.7	1.1	2.2	4.4	8.6
Unspecified liver disease	Partially attributable	0.4	0.4	0.8	1.7	6.5	11.1	12.5	16.0
heart failure	Partially attributable	1.0	1.0	1.3	1.1	5.2	14.6	35.6	120.8
Cholelithiasis	Partially attributable	1.3	1.3	4.9	6.4	27.1	60.7	121.6	200.5
Acute and chronic pancreatitis	Partially attributable	1.4	1.4	3.2	3.3	11.2	19.5	27.5	39.5
Psoriasis	Partially attributable	0.8	0.8	1.6	1.4	4.3	6.5	6.1	6.0
Spontaneous abortion	Partially attributable	0.0	0.0	0.0	0.0	0.0	0.0	0.0	0.0
Road traffic accidents - non pediastrian	Partially attributable	30.0	29.7	16.8	8.2	14.4	15.3	10.9	15.5
Pedestrian traffic accidents	Partially attributable	0.6	0.6	0.5	0.2	0.4	0.5	0.5	1.4
Water transport accidents	Partially attributable	0.2	0.2	0.3	0.2	0.2	0.4	0.2	0.2
Air/space transport accidents	Partially attributable	0.0	0.0	0.1	0.1	0.1	0.1	0.1	0.0
Fall injuries	Partially attributable	52.0	51.5	41.2	21.4	55.4	100.8	204.3	937.2
Work/machine injuries	Partially attributable	27.6	27.3	23.3	10.4	18.6	19.5	11.9	6.8
Firearm injuries	Partially attributable	1.7	1.7	0.6	0.2	0.4	0.3	0.2	0.1
Drowning	Partially attributable	0.1	0.1	0.0	0.0	0.1	0.1	0.1	0.3
Inhalation of gastric contents	Partially attributable	0.2	0.2	0.2	0.2	0.5	1.1	2.8	7.9
Fire injuries	Partially attributable	1.8	1.8	1.3	0.6	1.2	1.3	1.7	2.7
Accidental excessive cold	Partially attributable	0.0	0.0	0.0	0.0	0.1	0.2	0.2	1.4
Intentional self-harm	Partially attributable	28.5	28.2	25.5	11.9	18.0	13.8	9.9	15.5
Assault	Partially attributable	66.3	65.7	38.3	13.4	16.4	8.6	4.4	3.5

Table A17: Female SEC-adjusted alcohol-related morbidity rates (per 100,000) for England in 2005 for SEC1.1

Condition	Type	16-17	18-24	25-34	35-44	45-54	55-64	65-74	75+
Alcohol-induced pseudo-Cushing's syndrome	Wholly attributable	0.0	0.0	0.0	0.0	0.0	0.0	0.0	0.0
Mental and behavioural disorders due to use of alcohol	Wholly attributable	52.2	51.0	49.8	40.5	65.2	44.3	31.6	23.8
degeneration	Wholly attributable	0.0	0.0	0.0	0.1	0.3	0.3	0.2	0.1
Alcoholic polyneuropathy	Wholly attributable	0.0	0.0	0.1	0.0	0.2	0.1	0.1	0.1
Alcoholic myopathy	Wholly attributable	0.0	0.0	0.0	0.0	0.0	0.0	0.0	0.0
Alcoholic cardiomyopathy	Wholly attributable	0.0	0.0	0.0	0.0	0.1	0.1	0.0	0.1
Alcoholic gastritis	Wholly attributable	0.5	0.5	0.3	0.3	0.3	0.2	0.1	0.0
Alcoholic liver disease	Wholly attributable	0.2	0.2	2.3	4.5	12.8	12.6	10.5	3.9
Chronic pancreatitis	Wholly attributable	0.2	0.2	0.4	0.5	0.8	0.3	0.3	0.0
Ethanol poisoning	Wholly attributable	39.5	38.6	27.2	15.5	13.9	4.5	1.7	0.6
Methanol poisoning	Wholly attributable	0.0	0.0	0.0	0.0	0.0	0.0	0.0	0.0
Toxic effect of alcohol, unspecified	Wholly attributable	1.3	1.3	0.9	0.5	0.4	0.2	0.1	0.0
Accidental poisoning by exposure to alcohol	Wholly attributable	1.2	1.2	0.7	0.4	0.3	0.2	0.0	0.1
Malignant neoplasm of lip, oral cavity and pharynx	Partially attributable	0.3	0.3	0.8	0.9	3.6	5.8	7.9	8.7
Malignant neoplasm of oesophagus	Partially attributable	0.0	0.0	0.1	0.4	2.4	7.0	14.7	28.1
Malignant neoplasm of colon	Partially attributable	0.3	0.3	1.0	1.8	8.4	22.1	50.7	79.3
Malignant neoplasm of rectum	Partially attributable	0.1	0.1	0.6	0.9	4.3	10.3	20.7	27.7
Malignant neoplasm of liver and intrahepatic bile ducts	Partially attributable	0.2	0.2	0.2	0.2	1.0	2.1	4.7	7.2
Malignant neoplasm of larynx	Partially attributable	0.0	0.0	0.0	0.1	0.7	1.4	2.0	2.4
Malignant neoplasm of breast	Partially attributable	0.5	0.5	11.5	33.2	100.6	119.3	113.6	77.1
Diabetes mellitus (typeII)	Partially attributable	4.9	4.8	15.3	21.9	66.0	102.0	200.0	262.0
Epilepsy and status epilepticus	Partially attributable	58.7	57.3	64.6	33.6	50.9	53.9	70.1	103.2
Hypertensive diseases	Partially attributable	11.7	11.4	43.3	83.1	360.5	755.9	1543.7	2147.8
Ischaemic heart disease	Partially attributable	1.0	0.9	3.7	11.6	59.8	125.1	257.3	436.7
Cardiac arrhythmias	Partially attributable	11.7	11.5	17.7	13.5	41.1	114.9	401.6	1271.7
haemorrhagic stroke	Partially attributable	1.5	1.5	2.6	3.6	9.8	12.1	20.9	43.1
Ischaemic stroke	Partially attributable	1.0	1.0	2.8	3.4	12.0	27.6	86.6	254.5
Oesophageal varices	Partially attributable	0.5	0.5	0.7	0.6	2.0	3.3	5.3	3.9
Gastro-oesophageal laceration-haemorrhage syndrome	Partially attributable	4.1	4.0	2.4	0.8	1.0	1.4	2.5	4.8
Unspecified liver disease	Partially attributable	1.2	1.2	1.8	2.2	6.4	11.4	20.1	17.0
heart failure	Partially attributable	1.9	1.9	2.8	1.9	4.8	9.9	26.5	105.6
Cholelithiasis	Partially attributable	46.7	45.6	93.9	61.7	102.2	125.2	155.2	197.4
Acute and chronic pancreatitis	Partially attributable	8.0	7.8	11.3	7.2	13.2	17.3	24.4	32.6
Psoriasis	Partially attributable	5.1	5.0	6.1	2.9	4.4	5.1	4.7	4.7
Spontaneous abortion	Partially attributable	153.0	149.4	238.0	73.2	3.7	0.0	0.0	1.4
Road traffic accidents - non pediastrian	Partially attributable	30.5	29.8	18.7	6.5	8.2	7.5	8.1	10.7
Pedestrian traffic accidents	Partially attributable	0.9	0.8	0.5	0.2	0.4	0.6	0.5	1.1
Water transport accidents	Partially attributable	0.1	0.1	0.2	0.1	0.1	0.1	0.2	0.2
Air/space transport accidents	Partially attributable	0.0	0.0	0.1	0.0	0.0	0.0	0.0	0.0
Fall injuries	Partially attributable	68.6	67.0	70.3	36.8	74.0	130.9	314.6	1617.4
Work/machine injuries	Partially attributable	18.2	17.7	14.1	6.3	6.5	3.9	2.9	2.3
Firearm injuries	Partially attributable	0.4	0.4	0.1	0.1	0.0	0.1	0.0	0.0
Drowning	Partially attributable	0.1	0.1	0.1	0.1	0.0	0.1	0.1	0.2
Inhalation of gastric contents	Partially attributable	0.3	0.3	0.5	0.3	0.5	0.9	1.5	5.7
Fire injuries	Partially attributable	1.3	1.3	1.2	0.6	0.7	0.7	1.0	2.4
Accidental excessive cold	Partially attributable	0.0	0.0	0.0	0.0	0.0	0.0	0.2	1.5
Intentional self-harm	Partially attributable	150.4	146.8	78.9	38.2	35.5	17.0	12.2	13.1
Assault	Partially attributable	28.7	28.0	19.1	6.6	4.6	1.8	1.5	3.2

2. New sensitivity analyses

2.1. Probabilistic sensitivity analysis

In this analysis, probability distributions are fitted to the base case econometric modelling parameters that drive the impact of MUP policies. The pseudo-panel approach used to estimate price elasticities produces variance-covariance matrices for each of the beverage-specific models. For example, Table A18 shows the variance-covariance matrix for the off-trade beer base case model. Assuming conditions of multivariate normality, Cholesky decomposition can be used to sample alternative parameter estimates (from which own-price and cross-price elasticities can directly be derived). The model is then re-run with the new parameter estimates to generate fresh outcomes. The process is repeated a large number of times (30 here due to the time required for model runs, but ideally more) to produce a distribution of outcomes. From this, estimates of the 95% confidence interval around consumption reductions are obtained. The estimated confidence intervals are shown in Figure 2 of the main article.

2.2. Alternative price elasticity matrices

The price elasticity matrix is the key driver of policy impact; therefore, we investigate the robustness of our results to alternative methods of defining and deriving the matric. Four alternative sets of price elasticity matrices were used in sensitivity analyses. These are:

1. Own-price elasticities only (Table A19)
2. Significant own-price and cross-price elasticities only (Table A20)
3. Separate elasticity matrices for low vs. higher income groups (Tables A20 and A21)
4. Separate elasticity matrices for moderate vs. hazardous and harmful drinkers (Table A22 and A23)

All elasticity matrices were calculated using the pseudo-panel method outlined in the main article and described in more detail elsewhere.[5] For the income and consumption group-specific matrices, separate analyses are conducted for each group within the LCF and the matrices are then applied to individuals in the corresponding group within the GLF. Households are categorised as low income or higher income based on whether their equivalised household incomes are above or below 60% of the median equivalised household income. This threshold is the standard definition of relative poverty in the UK and uses equivalised household income to account for differences in levels of disposable income based on household composition.

Results of the sensitivity analyses are presented in Figures A1-A2 and support the conclusions of the main article which seem very robust to the use of alternative price elasticities. In each sensitivity analysis, consumption among moderate drinkers in all income groups is largely unaffected by the policy whereas an income trend is consistently seen in the effects for hazardous and, particularly, harmful drinkers. Similarly, as in the base case, spending changes remain small for moderate drinkers while for hazardous and harmful drinkers spending tends to decreases in lower income groups and increase in higher income groups. The main differences in results across the sensitivity analyses reflect that, compared to the base case elasticities, the low income and hazardous and harmful drinker

groups have larger price elasticities and the higher income and moderate drinker groups have smaller price elasticities. As such, under the income group-specific elasticities, the lowest income quintile reduce their consumption and decrease their spending by greater amounts compared to the base case while the higher income groups reduce their consumption by less and increase their spending by more. Similarly, under the consumption group-specific elasticities, moderate drinkers' reduce their consumption by less compared to the base case while hazardous and harmful drinkers reduce their consumption and consumption by more.

The results for the income and consumption group-specific elasticity matrices should be treated with caution as the underlying analyses violate assumptions of the pseudo-panel method and may not be robust. Specifically, it is recommended that each population subgroup, or panel member in the pseudo-panel, contains at least 100 survey respondents at each wave and this was not the case when deriving elasticity matrices for the low income or hazardous and harmful drinker populations.

Previous versions of SAPM used separate price elasticities for moderate vs. hazardous and harmful drinkers in the base case. These elasticities were calculated by pooling the cross-sectional waves of LCF data and conducting a three-stage least squares regression.[6] The pseudo-panel approach addresses several limitations identified with our previous approach (e.g. it analyses the data longitudinally and better addresses non-purchasers). Therefore, despite using a single elasticity matrix, we consider it to provide a more robust estimate of consumers' price responsiveness in England.

Table A18: Variance-covariance matrix for the off-trade beer model using the pseudo-panel approach

		Purchase									
		Off-beer	Off-cider	Off-wine	Off-spirit	Off-RTD	On-beer	On-cider	On-wine	On-spirit	On-RTD
Price	Off-beer	0.032									
	Off-cider	-0.004	0.008								
	Off-wine	-0.012	0.002	0.033							
	Off-spirit	-0.001	-0.003	-0.005	0.012						
	Off-RTD	0.000	0.000	0.000	0.000	0.002					
	On-beer	-0.004	0.000	-0.001	0.001	0.001	0.041				
	On-cider	-0.007	0.000	0.007	-0.001	0.000	-0.003	0.009			
	On-wine	0.004	0.001	-0.013	0.000	0.001	0.001	0.000	0.014		
	On-spirit	0.001	-0.004	-0.004	0.005	0.000	-0.002	-0.002	-0.004	0.014	
	On-RTD	0.000	0.000	0.002	0.001	0.000	-0.001	-0.001	-0.002	0.001	0.006

Table A19: P-values for base-case elasticity matrix (see Table 1 in main paper).

		Purchase									
		Off-beer	Off-cider	Off-wine	Off-spirits	Off-RTDs	On-beer	On-cider	On-wine	On-spirits	On-RTDs
Price	Off-beer	**0.000**	0.634	0.584	0.085	0.057	0.935	0.918	0.259	0.913	0.246
	Off-cider	0.472	**0.000**	0.115	0.282	0.326	0.348	0.662	0.437	0.299	0.271
	Off-wine	0.828	**0.037**	**0.019**	0.088	0.904	0.082	0.667	0.770	0.399	0.683
	Off-spirits	0.310	0.937	0.090	0.625	0.884	0.081	0.078	0.971	0.577	0.422
	Off-RTDs	0.332	0.156	0.873	0.165	**0.029**	0.157	0.626	0.347	**0.049**	0.563
	On-beer	0.467	0.504	0.574	0.905	0.124	**0.005**	0.200	**0.006**	**0.001**	0.816
	On-cider	0.281	0.640	0.583	0.881	0.080	0.782	**0.010**	0.510	**0.044**	0.238
	On-wine	0.097	0.667	0.270	0.851	0.769	0.130	0.906	**0.000**	0.897	0.063
	On-spirits	0.873	0.604	0.800	0.083	0.612	0.989	0.335	0.475	**0.000**	**0.014**
	On-RTDs	0.324	0.975	0.207	0.622	0.183	0.169	0.190	0.778	0.555	0.494

Note: Bold cells indicate p<0.05

Table A20: Estimated own-price elasticities of off- and on-trade beer, cider, wine, spirits and RTDs in the UK (excluding cross-price elasticities)

		Purchase									
		Off-beer	Off-cider	Off-wine	Off-spirits	Off-RTDs	On-beer	On-cider	On-wine	On-spirits	On-RTDs
Price	Off-beer	-0.980*									
	Off-cider		-1.268*								
	Off-wine			-0.384*							
	Off-spirits				-0.082						
	Off-RTDs					-0.585*					
	On-beer						-0.786*				
	On-cider							-0.591*			
	On-wine								-0.871*		
	On-spirits									-0.890*	
	On-RTDs										-0.187

Note:* p-value <0.05

Table A21: Estimated own- and cross-price elasticities of off- and on-trade beer, cider, wine, spirits and RTDs in the UK (excluding non-significant elasticities)

		Purchase									
		Off-beer	Off-cider	Off-wine	Off-spirits	Off-RTDs	On-beer	On-cider	On-wine	On-spirits	On-RTDs
Price	Off-beer	-0.980*									
	Off-cider		-1.268*								
	Off-wine		0.736*	-0.384*							
	Off-spirits										
	Off-RTDs					-0.585*				-0.179*	
	On-beer						-0.786*		1.042*	1.169*	
	On-cider							-0.591*		0.237*	
	On-wine								-0.871*		
	On-spirits									-0.890*	0.809*
	On-RTDs										

Note *: p-value <0.05

Table A22: Estimated own- and cross-price elasticities of off- and on-trade beer, cider, wine, spirits and RTDs in the UK for moderate drinkers

		Purchase									
		Off-beer	Off-cider	Off-wine	Off-spirits	Off-RTDs	On-beer	On-cider	On-wine	On-spirits	On-RTDs
Price	Off-beer	-0.439*	-0.353	0.324	-0.133	-0.611	-0.153	-0.493	0.290	-0.452	0.165
	Off-cider	-0.015	-0.677*	0.092	-0.066	-0.296	-0.036	0.126	0.132	-0.187	0.031
	Off-wine	-0.093	0.208	-0.418*	-0.455	0.270	-0.066	-0.217	-0.063	-0.051	0.327
	Off-spirits	-0.013	-0.193	0.066	-0.296*	0.416*	0.041	0.014	-0.010	0.014	0.084
	Off-RTDs	-0.099	-0.110	-0.080	0.421*	-0.355*	-0.131*	0.368*	-0.064	-0.048	-0.056
	On-beer	0.364	-0.933	-0.080	0.100	0.006	-0.380	-0.388	0.108	0.660	-0.214
	On-cider	-0.205	-0.408*	-0.176	-0.104	0.399	0.001	-0.484*	-0.176	-0.008	-0.093
	On-wine	0.133	0.043	0.315*	0.202	0.347	-0.073	0.053	-0.213	-0.162	-0.341
	On-spirits	-0.242	0.164	-0.046	0.268	0.116	-0.020	-0.030	0.309*	-0.183	0.154
	On-RTDs	-0.125	0.064	0.046	-0.330	0.654*	0.145	0.004	0.063	-0.163	0.229

Note *: p-value <0.05

Table A23: Estimated own- and cross-price elasticities of off- and on-trade beer, cider, wine, spirits and RTDs in the UK for hazardous and harmful drinkers

		Purchase									
		Off-beer	Off-cider	Off-wine	Off-spirits	Off-RTDs	On-beer	On-cider	On-wine	On-spirits	On-RTDs
Price	Off-beer	-1.094*	-0.120	-0.141	-0.085	-0.409	-0.058	1.638	0.164	0.818*	0.313
	Off-cider	0.006	-1.222*	-0.038	0.033	-0.131	-0.139	-0.382	0.047	-0.339	0.237
	Off-wine	0.443*	0.907	0.358	-0.128	1.508*	-0.272	0.463	0.273	-0.801	-0.523
	Off-spirits	-0.075	-0.165	-0.028	0.048	0.533	0.046	0.304	-0.134	0.127	-0.269
	Off-RTDs	-0.056	-0.180	0.043	0.030	-0.889*	0.064	0.033	0.035	-0.019	0.369
	On-beer	0.045	-0.621	-0.063	-0.246	0.321	-0.833*	1.049	0.263	0.816	-1.405
	On-cider	-0.054	0.268	-0.034	-0.121	0.078	0.019	-0.462	-0.031	0.384*	0.232
	On-wine	-0.102	0.243	0.048	-0.038	-1.055*	-0.067	0.321	0.052	0.539*	0.510
	On-spirits	-0.169	-0.540	-0.010	-0.199	-0.247	-0.385*	-0.479	0.009	-1.102*	0.563*
	On-RTDs	0.072	-0.155	-0.101	0.069	-0.366	-0.047	-0.395	0.049	-0.128	-0.800*

Note *: p-value <0.05

Table A24: Estimated own- and cross-price elasticities of off- and on-trade beer, cider, wine, spirits and RTDs in the UK for low income drinkers

		Purchase									
		Off-beer	Off-cider	Off-wine	Off-spirits	Off-RTDs	On-beer	On-cider	On-wine	On-spirits	On-RTDs
Price	Off-beer	-0.883*	-0.443	-0.350	-0.186	-2.677*	-0.306	-1.011	-0.820*	-1.245*	0.594
	Off-cider	0.191	-1.751*	0.024	-0.361	-0.588	-0.050	0.085	-0.117	-0.290	-0.523*
	Off-wine	-0.094	0.483	-0.472	-0.111	0.449	0.037	-0.851	0.436*	-0.778	0.210
	Off-spirits	-0.024	0.480	0.335	-0.256	0.868	0.307*	1.057*	0.079	0.310	-0.093
	Off-RTDs	-0.032	-0.416*	0.009	-0.062	0.204	-0.081	0.293	0.021	-0.133	-0.036
	On-beer	0.041	-0.311	0.865*	-0.703	1.456	-0.504	3.785*	0.903	1.698*	-3.893*
	On-cider	-0.169	0.311	0.008	0.223	-0.625	0.242	0.611	-0.208	0.057	0.058
	On-wine	-0.311	-0.427	0.080	-0.405	0.773	0.006	-1.129	-0.664*	1.021	0.088
	On-spirits	0.111	0.295	0.029	-0.152	1.176	0.166	0.063	0.324	-1.108*	1.126*
	On-RTDs	0.223	0.241	-0.014	-0.305	-0.532	0.187	-0.081	0.373*	0.382	0.543

Note *: p-value <0.05

Table A25: Estimated own- and cross-price elasticities of off- and on-trade beer, cider, wine, spirits and RTDs in the UK for higher income drinkers

		Purchase									
		Off-beer	Off-cider	Off-wine	Off-spirits	Off-RTDs	On-beer	On-cider	On-wine	On-spirits	On-RTDs
Price	Off-beer	-0.914*	-0.033	0.194	-0.448	-0.923	0.044	0.110	0.419	0.311	0.673
	Off-cider	0.046	-1.217*	0.170	-0.079	-0.159	-0.060	0.085	0.093	-0.140	-0.178
	Off-wine	-0.017	0.775	-0.417*	0.359	-0.138	-0.331	-0.020	-0.097	-0.085	0.019
	Off-spirits	0.129	-0.046	0.133	0.098	-0.077	0.142	0.312	0.096	0.061	0.341
	Off-RTDs	-0.024	-0.110	0.004	0.086	-0.730*	-0.058	-0.018	0.058	-0.202	0.180
	On-beer	0.157	-0.317	0.024	0.050	0.694	-0.897*	0.563	0.908*	1.079*	0.297
	On-cider	-0.117	0.041	0.045	-0.001	0.367	0.031	-0.797*	0.091	0.232	0.245
	On-wine	-0.188	0.183	-0.203	-0.021	-0.045	-0.275	0.053	-0.858*	-0.105	-0.288
	On-spirits	0.025	-0.212	-0.036	-0.347	-0.386	-0.039	-0.499	0.057	-0.783*	0.898*
	On-RTDs	0.033	-0.104	-0.113	0.021	0.429	0.094	-0.510	-0.073	-0.172	-0.236

Note *: p-value <0.05

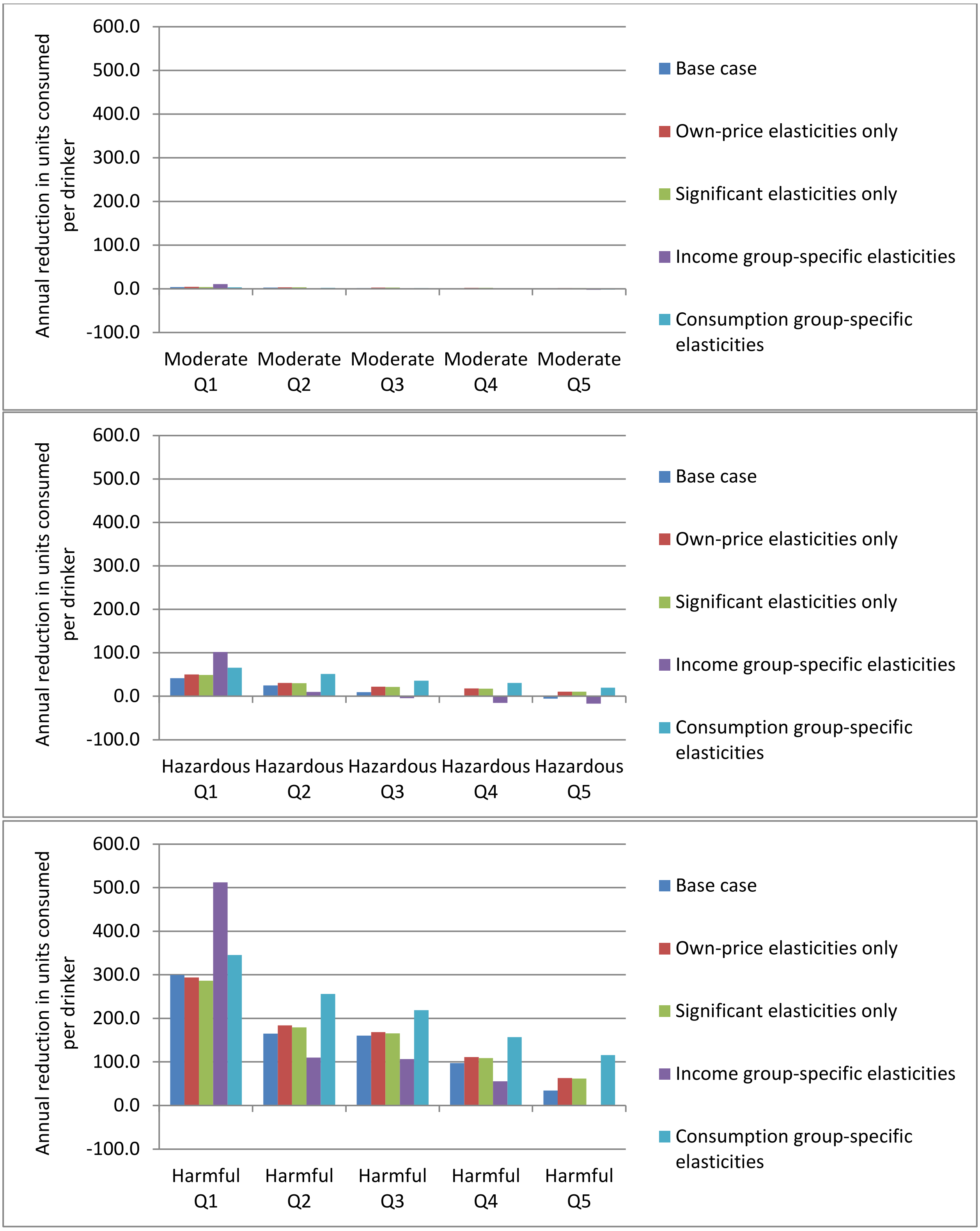

Figure A1: Annual reductions in units consumed per drinker under different price elasticities for moderate (top), hazardous (middle) and harmful (drinkers) by income quintile (Q1 lowest – Q5 highest).

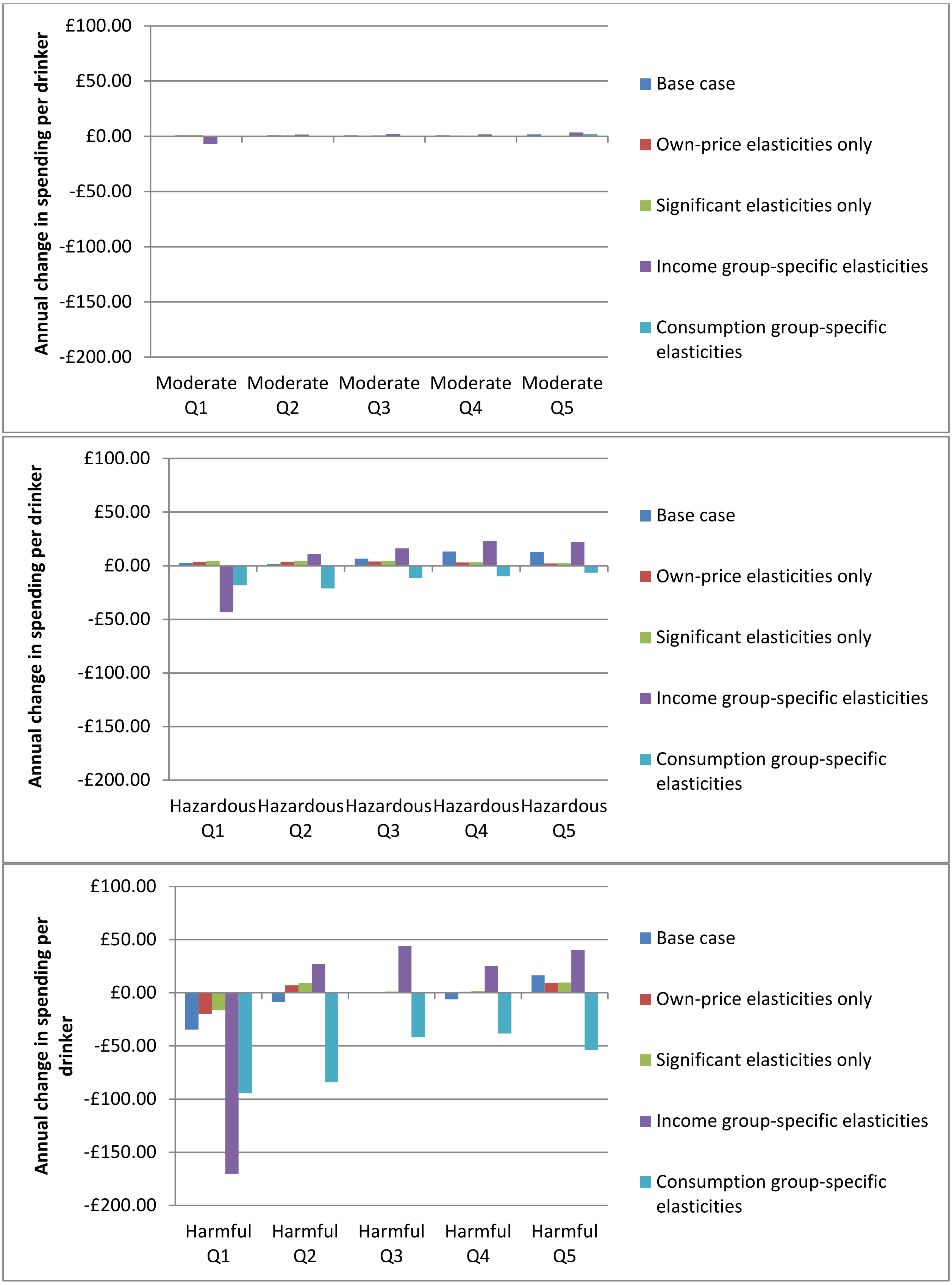

Figure A2: Annual change in spending per drinker under different price elasticities for moderate (top), hazardous (middle) and harmful (drinkers) by income quintile (Q1 lowest – Q5 highest).

2.3. Alternative price thresholds

Previous analyses have demonstrated that the scale of impact from minimum unit pricing (MUP) policies increases as the price threshold is raised. To examine whether our conclusions were robust to higher and lower price thresholds, we repeated the income group analysis for MUP thresholds of 40p and 50p. The results of the consumption and spending impacts are presented in Table A24 and show that, as expected, the size of the impact from the policy increases as the price threshold increases; however, the patterning of the results across groups and the conclusions which can be drawn from this remain the same. For all price thresholds, impacts on spending and consumption among moderate drinkers are small in each income group. Income gradients for consumption and spending impacts remain for hazardous and harmful drinkers and policy impacts become more substantive for higher income hazardous and harmful drinkers at higher price thresholds. In general, fewer spending reductions and more spending increases are seen for higher price thresholds.

Table A26: Estimated changes in alcohol consumption and spending under alternative minimum unit pricing thresholds by income and consumption group.

	Population	Q1 (low)	Q2	Q3	Q4	Q5 (high)
				All drinkers		
Change in consumption (yearly units per drinker) 40p	-7.2	-18.6	-9.3	-7.4	-3.1	-0.3
Change in consumption (yearly units per drinker) 45p	-11·7	-29·3	-15·1	-12·4	-5·5	-0·8
Change in consumption (yearly units per drinker) 50p	-18.0	-42.8	-23.0	-19.6	-9.1	-1.9
Change in spending (£ per drinker per year) 40p	-£0.08	-£2.94	-£1.28	-£0.31	£0.75	£2.54
Change in spending (£ per drinker per year) 45p	£2·12	-£1·89	£0·11	£1·84	£3·22	£6·10
Change in spending (£ per drinker per year) 50p	£6.38	£0.48	£3.19	£5.73	£7.92	£12.72
				Moderate drinkers		
Change in consumption (yearly units per drinker) 40p	-0.9	-2.4	-1.8	-0.9	-0.5	0.7
Change in consumption (yearly units per drinker) 45p	-1·6	-3·8	-2·8	-1·6	-0·8	0·8
Change in consumption (yearly units per drinker) 50p	-2.7	-5.9	-4.5	-2.8	-1.4	0.8
Change in spending (£ per drinker per year) 40p	£0.02	-£0.64	-£0.38	-£0.03	£0.12	£0.98
Change in spending (£ per drinker per year) 45p	£0·78	£0·04	£0·34	£0·79	£0·85	£1·86
Change in spending (£ per drinker per year) 50p	£2.25	£1.32	£1.61	£2.29	£2.37	£3.60
				Hazardous drinkers		
Change in consumption (yearly units per drinker) 40p	-4.5	-25.2	-14.3	-4.8	2.3	4.5
Change in consumption (yearly units per drinker) 45p	-9·1	-41·7	-24·8	-9·5	1·7	5·4
Change in consumption (yearly units per drinker) 50p	-16.4	-64.9	-39.5	-17.9	-1.4	6.3
Change in spending (£ per drinker per year) 40p	£2.91	-£2.53	-£2.07	£1.01	£5.97	£6.81
Change in spending (£ per drinker per year) 45p	£8·67	£2·60	£1·56	£6·80	£13·19	£12·89
Change in spending (£ per drinker per year) 50p	£19.26	£11.01	£9.69	£17.45	£25.80	£24.17
				Harmful drinkers		
Change in consumption (yearly units per drinker) 40p	-88.4	-193.2	-105.1	-99.3	-61.0	-24.2
Change in consumption (yearly units per drinker) 45p	-138·2	-299·.8	-165·0	-160·3	-97·1	-34·3
Change in consumption (yearly units per drinker) 50p	-201.1	-424.0	-241.4	-240.0	-145.5	-50.0
Change in spending (£ per drinker per year) 40p	-£11.04	-£30.63	-£11.92	-£7.88	-£12.26	-£0.39
Change in spending (£ per drinker per year) 45p	-£4·01	-£34·63	-£8·67	-£0·11	-£6·08	£16·35
Change in spending (£ per drinker per year) 50p	£11.78	-£33.15	£2.99	£13.69	£8.47	£45.37

The impact of alternative MUP threshold health harms are presented in Table A25 and similarly show policy impacts increase across all socioeconomic groups as the price threshold increases. However, the pattern of results remains the same. Although the distribution of harm reductions is marginally more equal across groups at higher price thresholds, the vast majority of harm reductions remain in the routine and manual worker household group.

Table A27: Estimated changes in health outcomes at year 10 under alternative minimum unit pricing thresholds by income and consumption group.

		Population	Long-term unemployed	Routine /manual	Intermediate	Managerial/ professional
Annual changes in Year 10	Deaths 40p	-5.6%	-7.3%	-8.4%	-2.6%	-1.9%
	Deaths 45p	-8·6%	-11.8%	-13·8%	-5·0%	-3·0%
	Deaths 50p	-12.9%	-17.7%	-18.3%	-7.5%	-5.3%
	Hospital admissions ('000s) 40p	-2.5%	-2.3%	-4.1%	-0.9%	-0.6%
	Hospital admissions ('000s) 45p	-3·7%	-3.8%	-6·4%	-1·6%	-0·9%
	Hospital admissions ('000s) 50p	-5.4%	-5.6%	-8.6%	-2.5%	-1.6%
	QALYs ('000s) 40p	-2.2%	-3.0%	-3.1%	-0.9%	-0.4%
	QALYs ('000s) 45p	-3·4%	-4.8%	-5·4%	-1·7%	-0·8%
	QALYs ('000s) 50p	-5.2%	-7.3%	-7.5%	-2.7%	-1.5%
	% of deaths reduction 40p	100.0%	2.5%	80.7%	7.7%	9.2%
	% of deaths reduction 45p	100·0%	2.6%	79·2%	8·8%	12·0%
	% of deaths reduction 50p	100.0%	2.6%	76.4%	9.6%	11.5%
	% of hospital admissions 40p	100.0%	2.4%	84.6%	6.5%	6.5%
	% of hospital admissions 45p	100·0%	2.5%	83·8%	7·5%	8·7%
	% of hospital admissions 50p	100.0%	2.6%	80.6%	8.3%	8.5%
	% of total QALYs saved 40p	100.0%	3.4%	85.1%	6.8%	4.7%
	% of total QALYs saved 45p	100·0%	3.5%	84·9%	8·3%	6·8%
	% of total QALYs saved 50p	100.0%	3.6%	80.7%	9.0%	6.8%
Cumulative discounted cost changes: Years 1-10	Total cost savings 40p	-2.2%	-2.8%	-3.3%	-0.8%	-0.4%
	Total cost savings 45p	-3·3%	-4.5%	-5·3%	-1·6%	-0·7%
	Total cost savings 50p	-5.1%	-6.8%	-7.3%	-2.5%	-1.4%

2.4. Never worked and long-term unemployed - NS-SEC Group 8

The NS-SEC socioeconomic status classificatory system has eight groups, the last of which is the never worked and long-term unemployed (NS-SEC8). Our source data for alcohol-related mortality estimates by NS-SEC group provides no data for NS-SEC8.[3] After examining the relationship between income quintile and NS-SEC group membership (see Table A26), we concluded that the NS-SEC8 group typically has a low income. Therefore, in the base case, they are assigned to the next lowest NS-SEC group where mortality data is available (NS-SEC7). In this sensitivity analysis, we re-run the analysis under the more conservative assumption that NS-SEC8 have the average alcohol-related mortality rate for the population.

Model results comparing estimated impacts on health harms in the base case and the sensitivity analysis are shown in Table A27 and suggest reallocation of NS-SEC8 makes only marginal differences to the results.

Table A28: Relationship between income quintile and NS-SEC groups

	Quintile 1 (low)	Quintile 2	Quintile 3	Quintile 4	Quintile 5 (High)	All quintiles
NS-SEC 1.1	4.2%	7.2%	10.4%	24.8%	53.4%	100.0%
NS-SEC 1.2	7.5%	5.4%	12.2%	24.3%	50.6%	100.0%
NS-SEC 2	8.1%	13.3%	20.9%	28.0%	29.6%	100.0%
NS-SEC 3	16.9%	24.5%	23.5%	21.9%	13.1%	100.0%
NS-SEC 4	33.7%	24.5%	23.1%	9.7%	9.0%	100.0%
NS-SEC 5	13.4%	25.7%	27.8%	24.1%	9.0%	100.0%
NS-SEC 6	33.0%	29.1%	22.6%	12.2%	3.2%	100.1%
NS-SEC 7	34.4%	32.3%	19.0%	10.8%	3.5%	100.0%
NS-SEC 8	67.8%	15.8%	9.0%	3.1%	4.2%	100.0%

Table A29: Health outcome changes at year 10 for a 45p MUP by socioeconomic group with different assignment of NS-SEC8

		Population	Long-term unemployed	Routine /manual	Intermediate	Managerial/ professional
Annual changes in Year 10	Deaths (BC)	-8.9%	-	-12.7%	-4.6%	-3.3%
	Deaths (SA)	-8.8%	11.8%	-12.8%	-4.6%	-3.3%
	Hospital admissions (‘000s) (BC)	-3.9%	-	-6.2%	-1.6%	-1.0%
	Hospital admissions (‘000s) (SA)	-3.8%	-3.8%	-6.2%	-1.6%	-1.0%
	QALYs (‘000s) (BC)	-3.6%	-	-5.2%	-1.6%	-0.8%
	QALYs (‘000s) (SA)	-3.5%	-4.8%	-5.2%	-1.6%	-0.8%
	% of deaths reduction (BC)	100·0%	-	81.8%	8.3%	9.9%
	% of deaths reduction (SA)	100.0%	2.6%	78.5%	8.6%	10.3%
	% of hospital admissions (BC)	100·0%	-	86.0%	7.0%	7.0%
	% of hospital admissions (SA)	100.0%	2.5%	82.7%	7.4%	7.4%
	% of total QALYs saved (BC)	100·0%	-	87.1%	7.5%	5.4%
	% of total QALYs saved (SA)	100.0%	3.5%	82.9%	7.9%	5.7%
Cumulative discounted cost change Years 1-10	Total cost savings (BC)	-3.5%	-	-5.1%	-1.5%	-0.8%
	Total cost savings (SA)	-3.5%	-4.8%	-5.2%	-1.6%	-0.8%

BC: Base case; SA: Sensitivity analysis.

3. Previous sensitivity analyses

SAPM was originally developed in 2008 and has been regularly updated and developed since as well as being adapted to appraise policy impacts in other countries. During this period, a range of sensitivity analyses have been conducted on key aspects of uncertainty in the underlying evidence base and datasets. Two of these are reproduced here from earlier technical reports as they are instructive for understanding the implications of uncertainties in the present analysis.

3.1. Effects of accounting for underestimation of alcohol consumption in self-report population surveys (source: Scottish adaptation of SAPM, second update)[7]

Alcohol consumption data from self-report population surveys are known to substantially underestimate a population's total consumption compared to more reliable tax or sales data.[8] However, survey data are essential for the types of subgroup analyses conducted using SAPM. We have developed a method for adjusting survey-based estimates of a population's consumption distribution to account for various known biases. The full method is described elsewhere; [8] in brief, the main steps are:

- Simulating additional survey respondents for missing populations (e.g. the homeless, prisoners);
- Adjustment of survey weights to account for key under-represented populations (e.g. students and dependent drinkers);
- Adjustments to individual consumption data to account for other biases (e.g. assumed size of self-poured drinks);
- Calibration of the revised consumption distribution to aggregated sales data using the gamma function.

When applied to the Scottish adaptation of SAPM, this method increased estimates of alcohol consumption derived from the Scottish Health Survey, the underlying consumption survey, from an average of 11.5 units per person per week to 19.6 units. The largest increases were due to underrepresentation of dependent drinkers within the survey (increased mean consumption by 4.7 units) and accounting for evidence showing drinkers typically under-estimate the size of self-poured spirits consumed in the off-trade (increased mean consumption by 3.1 units).

The impact of using the adjusted consumption distribution to model the effects of a 45p MUP in Scotland are summarised in Table A28 (full results available in the original report[7]). The results show accounting for underestimation of consumption leads to substantially larger estimated reductions in consumption, deaths, hospital admissions and associated costs.

3.2. Effects of making alternative assumption regarding who consumes purchased alcohol (source: version 2.0 of English SAPM)[9]

Estimates of alcohol purchasing and alcohol consumption for the English population are not available in a single survey and thus two surveys are combined to estimate mean and peak consumption levels and preferences for price point, beverage type and purchase location for

each population subgroup. Differences in survey design mean we assume purchasing in the two week LCF diary is equivalent to mean consumption by the purchaser. This assumption may introduce multiple errors and a detailed comparison of the LCF purchasing volumes for different beverage types compared to GLF consumption of those beverages suggests discrepancies between purchase and consumption, particularly for females aged 35+. To examine the impact of this, a sensitivity analysis was performed using version 2.0 of the English SAPM. Methods and results are reported in full in the original report to NICE.[9]

In brief, alcohol purchases in the LCF were reallocated using a stochastic heuristic which yields an improved match between the LCF and GLF consumption data:

> *"For women whose beer or spirit consumption exceeds 30% in the LCF data, 70% of their off-trade beer transactions and 40% of their off-trade spirit transactions are randomly reallocated to men. For older women (age 25 and older) whose beer or spirit consumption exceeds 30%, 4% of their off-trade wine transactions are reallocated to younger women"*

The results of this sensitivity analysis are summarised in Table A29 and suggest reallocations of consumption lead to larger estimates of policy impact on alcohol-related harm and costs which are primarily driven by substantially bigger reductions in consumption among harmful drinkers.

Table A30: Estimated impacts of a 45p MUP in Scotland with and without adjustment for underestimation of consumption[b]

	Annual reduction in units consumed per drinker				Annual harm reduction in year 10		Cumulative discounted cost changes (inc. QALYs): Years 1-10
	Population	Moderate	Hazardous	Harmful	Deaths	Hospital admissions	
Base case	-25	-5	-38	-278	-196	-4,100	£371m
Adjusted consumption distribution	-51	-10	-60	-352	-264	-5,700	£495m

[b] *Results taken from previous analyses using version 2.2 of the Scottish adaptation of SAPM*[7]

Table A31: Estimated impacts of a 45p MUP in England with and without alternative assumptions regarding who consumes purchased alcohol[c]

	Annual reduction in units consumed per drinker				Annual harm reduction in year 10		Cumulative discounted cost changes (inc. QALYs): Years 1-10
	Population	Moderate	Hazardous	Harmful	Deaths	Hospital admissions	
Base case	-35	-7	-45	-253	-2,040	-66,200	£2,387m
Alternative assumption	-39	-6	-47	-300	-2,366	-75,000	£2,653m

[c] *Results taken from previous analyses using version 2.0 of the English SAPM*[9]

4. References

Reference List

1 Jones, L, Bellis, MA, Dedman, D, Sumnall, H, Tocque, K. Alcohol-attributable fractions for England: alcohol-attributable mortality and hospital admissions. Liverpool: Liverpool John Moores University, Centre for Public Health; 2008.

2 Office for National Statistics. Population Estimates for UK, England and Wales, Scotland and Northern Ireland, mid 2005.
2010 http://www.ons.gov.uk/ons/publications/re-reference-tables.html?edition=tcm%3A77-213624 accessed 4 October 2013.

3 Siegler V, Al-Hamad A, Johnson B, Wells C, Sheron N. Social inequalities in alcohol-related adult mortality by National Statistics Socio-economic Classification, England and Wales, 2001-03. *Health Statistics Quarterly* 2011; **50**: 1-36.

4 Office for National Statistics. Population Estimates for UK, England and Wales, Scotland and Northern Ireland, Mid-2010. 2011

5 Meng, Y, Brennan, A, Purshouse, R, Hill-MacManus, D, Angus, C, Holmes, J, Meier, P. Estimation of own and cross price elasticities of alcohol demand in the UK: A pseudo-panel approach using the Live Costing and Food Survey 2001 to 2009. Sheffield: ScHARR, University of Sheffield; 2013. Health Economics and Decisions Science Discussion Paper DP 13/11.

6 Brennan, A, Purshouse, RC, Taylor, K, Rafia, R, Meier, P, Booth, A, et al. Independent review of the effects of alcohol pricing and promotion: part B. Results from the Sheffield Alcohol Policy Model. Sheffield: University of Sheffield; 2008.

7 Meng, Y, Hill-MacManus, D, Brennan, A, Meier, P. Model-based appraisal of alcohol minimum pricing and off-licensed trade discount bans in Scotland using the Sheffield Alcohol Policy Model (v 2): Second update based on newly available data. Sheffield: ScHARR, University of Sheffield; 2012.

8 Meier PS, Meng Y, Holmes J et al. Adjusting for unrecorded consumption in survey and per capita sales data: Quantification of impact on gender- and age-specific alcohol attributable fractions for oral and pharyngeal cancers in Great Britain. *ALCOHOL ALCOHOLISM* 2013 January; **48**: 241-9.

9 Purshouse, RC, Brennan, A, Latimer, N, Meng, Y, Rafia, R, Jackson, R. Modelling to assess the effectiveness and cost-effectiveness of public health related strategies and interventions to reduce alcohol attributable harm in England using the Sheffield Alcohol Policy Model version 2.0: Report to the NICE Public Health Programme Development Group. Sheffield: University of Sheffield; 2009.
